# Consistent diurnal pattern of leaf respiration in the light among contrasting species and climates

**DOI:** 10.1111/nph.18330

**Published:** 2022-07-12

**Authors:** Andreas H. Faber, Kevin L. Griffin, Mark G. Tjoelker, Majken Pagter, Jinyan Yang, Dan Bruhn

**Affiliations:** ^1^ Department of Chemistry and Bioscience Aalborg University Fredrik Bajers Vej 7H 9220 Aalborg Denmark; ^2^ Department of Earth and Environmental Sciences Columbia University Palisades NY 10964 USA; ^3^ Department of Ecology, Evolution and Environmental Biology Columbia University New York NY 10027 USA; ^4^ Lamont‐Doherty Earth Observatory Columbia University Palisades NY 10964 USA; ^5^ Hawkesbury Institute for the Environment Western Sydney University Penrith NSW 2750 Australia

**Keywords:** dark respiration, diurnal rhythm, diurnal variation, Kok method, leaf respiration, light respiration, plant functional types

## Abstract

- Leaf daytime respiration (leaf respiration in the light, *R*_L_) is often assumed to constitute a fixed fraction of leaf dark respiration (*R*_D_) (i.e. a fixed light inhibition of respiration (*R*_D_)) and vary diurnally due to temperature fluctuations.
- These assumptions were tested by measuring *R*_L_, *R*_D_ and the light inhibition of *R*_D_ in the field at a constant temperature using the Kok method. Measurements were conducted diurnally on 21 different species: 13 deciduous, four evergreen and four herbaceous from humid continental and humid subtropical climates.
- *R*_L_ and *R*_D_ showed significant diurnal variations and the diurnal pattern differed in trajectory and magnitude between climates, but not between plant functional types (PFTs). The light inhibition of *R*_D_ varied diurnally and differed between climates and in trajectory between PFTs.
- The results highlight the entrainment of leaf daytime respiration to the diurnal cycle and that time of day should be accounted for in studies seeking to examine the environmental and biological drivers of leaf daytime respiration.

## Introduction

Terrestrial plants are estimated to fix 120 Gt carbon (C) every year through photosynthesis and roughly 30 Gt C is emitted to the atmosphere through leaf respiration (Prentice *et al*., 2001). This C efflux is approximately three times larger than current emissions from burning of fossil fuels globally (Canadell *et al*., 2007; Le Quéré *et al*., 2009; Friedlingstein *et al*., 2020). However, current modelling of leaf respiration C fluxes is considered inadequate, leading to uncertain estimates of future climate and vegetation dynamics (Gifford, 2003; Leuzinger & Thomas, 2011; Huntingford *et al*., 2013; Smith & Dukes, 2013; Lombardozzi *et al*., 2015).

Inadequate representation of leaf respiration in current modelling approaches is related to incomplete understanding of the environmental and biological controls of leaf daytime respiration (Kruse *et al*., 2011; Searle *et al*., 2011; Huntingford *et al*., 2013; Kornfeld *et al*., 2013; Tcherkez *et al*., 2017a,b). Leaf respiration is often measured during the day using darkened chambers (*R*_D_) (Atkin *et al*., 2015). However, rates of leaf respiration in the light (*R*_L_) are often substantially lower than those in darkness (Amthor & Baldocchi, 2001; Janssens *et al*., 2001; Morgenstern *et al*., 2004; Wohlfahrt *et al*., 2005; Bruhn *et al*., 2011; Bathellier *et al*., 2017). Failure to consider this light inhibition of respiration (*R*_D_) can lead to overestimates of daily respiratory fluxes in individual leaves (Atkin *et al*., 2006), and thus have implications for our understanding of how environmental and biological factors drive leaf daytime respiration.

The light inhibition of *R*_D_ can depend on temperature (Atkin *et al*., 2000, 2006; Griffin & Turnbull, 2013; Way & Yamori, 2014), drought (Ayub *et al*., 2011; Crous *et al*., 2012; Sperlich *et al*., 2016), CO_2_ (Shapiro *et al*., 2004; Ayub *et al*., 2014), long‐term growth temperature (Heskel *et al*., 2014; McLaughlin *et al*., 2014), soil nutrient availability (Heskel *et al*., 2012; Atkin *et al*., 2013), season (Way *et al*., 2015) and plant functional type (PFT) (Heskel *et al*., 2012, 2014; Crous *et al*., 2017a). Some approaches account for the light inhibition of *R*_D_ by assuming *R*_L_ constitutes a fixed fraction of *R*_D_. For example, the terrestrial biosphere model (TBM) Joint UK Land Environmental Simulator (JULES) model assumes *R*_D_ is 30% inhibited when light is available (Cox, 2001; Clark *et al*., 2011). Although several studies demonstrate a mean light inhibition of *c*. 30% (Budde & Randall, 1990; Tcherkez *et al*., 2005, 2009, 2012b, 2017a; Buckley & Adams, 2011; Heskel *et al*., 2013; Kroner & Way, 2016), the light inhibition of *R*_D_ can vary between 0 and 100% (Atkin *et al*., 2006; Zaragoza‐Castells *et al*., 2007; Crous *et al*., 2012; Heskel *et al*., 2013; Way *et al*., 2019) and occasionally *R*_L_ even exceeds *R*_D_ (Zaragoza‐Castells *et al*., 2007; Crous *et al*., 2017a). Studying temporal patterns of *R*_L_ and *R*_D_ could shed light on the validity of the assumption of a fixed proportion of light inhibition of *R*_D_ and provide further insight into daytime leaf respiration.

Leaf respiration varies diurnally due to temperature fluctuations and is a key driver in modelled respiration (Running & Coughlan, 1988; Raich *et al*., 1991; Melillo *et al*., 1993; Cox, 2001; Clark *et al*., 2011; Oleson *et al*., 2013). However, leaf respiration may also be influenced by antecedent conditions. The amount of substrate available for respiration often directly relates to light intensity and photosynthesis (Högberg & Read, 2006). Substrate supply and demand processes for respiration can vary within hours depending on the environment (Trumbore, 2006; Hagedorn *et al*., 2016; O'Leary *et al*., 2019). Leaf respiration may also be under circadian regulation as protein expressions of enzymes central to respiration show diurnal rhythmicity (Wijnen & Young, 2006) and some indirect evidence, provided by statistical filtering techniques, shows that daytime net ecosystem CO_2_ exchange is affected by circadian regulation (Doughty *et al*., 2006; Resco de Dios *et al*., 2012).

If leaf respiration is under circadian regulation, and/or is dependent on previous environmental and leaf physiological conditions, leaf respiration will depend on the time of day even if the temperature is held constant. In addition, as *R*_L_ and *R*_D_ often respond differently to changes in the growth environment (Atkin *et al*., 2005; Zaragoza‐Castells *et al*., 2007; McLaughlin *et al*., 2014; Kroner & Way, 2016; Crous *et al*., 2017b), time of day could affect *R*_L_ and *R*_D_ differently. Given that the environment differs significantly between climates, and that rhythms in plant physiological processes are entrained by environmental cues such as light and temperature (Resco de Dios & Gessler, 2018), the effect of time of day on leaf respiration may vary between climates.

Respiratory fluxes differ among cooccurring species and PFTs under field conditions (Bolstad *et al*., 2003; Tjoelker *et al*., 2005; Turnbull *et al*., 2005; Heskel *et al*., 2012, 2014; Slot *et al*., 2013; Crous *et al*., 2017a) and under controlled environments (Reich *et al*., 1998; Loveys *et al*., 2003; Xiang *et al*., 2013), suggesting strong genetic control of respiratory fluxes. Hence, effects of time of day on *R*_L_, *R*_D_ and the light inhibition of *R*_D_ may be a function of PFTs and species. The consequences of time of day on leaf respiration and the magnitude of effects are currently unknown and could potentially be an important source of variation in leaf respiration. If true, time of day should be accounted for in measurements and models of respiration.

The aim of this study was to examine whether leaf daytime *R*_D_, *R*_L_ and the light inhibition of *R*_D_ exhibit diurnal variation when measured at a constant temperature in the field, and to determine whether this diurnal variation differs between climates and PFTs. The measurements were conducted using the Kok method (Kok, 1948) on 21 different species, including 13 deciduous, four evergreen and four herbaceous species from two contrasting climates: a humid continental and a humid subtropical. The validity of the Kok method as a reliable estimator of *R*_L_ has been under much debate (Buckley *et al*., 2017; Farquhar & Busch, 2017; Gauthier *et al*., 2020; Yin *et al*., 2020) as estimates of *R*_L_ using the method are influenced by increasing CO_2_ concentrations at the sites of carboxylation (*C*_c_), caused by decreasing stomatal conductance (*g*_s_) and mesophyll conductance (*g*_m_) at decreasing irradiance levels, and by changes in the quantum yield (ϕPsII) (Yin *et al*., 2011; Farquhar & Busch, 2017). However, the Kok method is currently viewed as a proxy for *R*_L_ (Gauthier *et al*., 2020; Yin *et al*., 2020), and was used here because of its field applicability compared to other methods, and because over 800 published papers have used or cited the method, which is *c*. 40% of all studies involved with daytime respiration (Tcherkez *et al*., 2017a). It was hypothesized that: the basal rate of *R*_L_, *R*_D_ and the light inhibition of *R*_D_, estimated with the Kok method, exhibit diurnal variations when measured at constant leaf temperature in the field; and the diurnal variations of the basal rate of *R*_L_, *R*_D_ and the light inhibition of *R*_D_, estimated with the Kok method, are different between climates and PFTs.

## Materials and Methods

### Study sites and climate conditions

The study took place in Denmark and Australia from 2019 to 2021. The measurements conducted in Denmark were collected from 31 August 2019 to 12 October 2019 in a mixed deciduous forest in northern Jutland (56°45′32″N, 10°12′4″E, 8 m above sea level (asl)) and again from 30 July 2021 to 30 August 2021 in another nearby mixed deciduous forest (56°40′59″N, 10°10′34″E, 20 m asl). The soil is a glacial outwash plain at both locations. In Australia, the measurements were conducted at the Hawkesbury Forest Experiment (HFE) site in Richmond, New South Wales (33°36′40″S, 150°44′26.5″E, 30 m asl) from 11 January 2020 to 29 February 2020 where the soil consists of a low‐fertility sandy loam (Drake *et al*., 2016). The climate at the Danish study sites is humid continental while the climate at the Australian study site is humid subtropical. Precipitation and temperature data from 2011 to 2021 and during the time of data collection can be found in the supplementary site description for the region covering the Danish study sites (Supporting Information Notes S1 Fig. A–B and G–J, respectively) and the study site in Australia (Notes S1 Fig. C–D and E–F, respectively). In total, 18 mm of rain fell in the summer period from the start of November 2019 to start of January 2020 at the study site in Australia, resulting in a significant dry period before the study period. Watering with cleaned wastewater every fourth day occurred at the study site in Australia for a subset of the measured species (Table S1).

### Species selection

Twenty‐one species in total, representing three PFTs, were included in the study: 13 deciduous tree species, four evergreen tree species and four herbaceous species, 10 of which were measured in Denmark, and the remainder in Australia (Table S1). For each species, measurements were only performed on individuals which on clear days were exposed to direct sunlight throughout most of the day.

### Leaf gas exchange measurements

Measurements of leaf net CO_2_ exchange (*A*_net_, μmol CO_2_ m^−^^2^ s^−1^), stomatal conductance for water vapour (*g*_sw_, mol m^−2^ s^−1^) and respiration in the dark were conducted on newly developed, fully expanded sun‐adapted leaves 0.1–2 m above ground level. Each leaf was measured 3–11 times throughout a single day, usually before sunrise until after sunset with a period of 35 min to 1 h in between each measurement. The measurements were conducted on at least four replicate plants (one leaf per plant) of each species under varying weather conditions (variation in precipitation, cloud cover, wind and temperature). The leaves within species were selected based on morphological similarity.

The measurements were conducted using a Li‐Cor 6800 portable photosynthesis system (Li‐Cor, Lincoln, NE, USA) with a 6 cm^2^ leaf chamber and a 6800‐01A fluorometer set to 69% red and 31% blue light. On each measurement day, leaf temperature was preset to a constant temperature (*T*_o_). For the measurements conducted in 2019 in Denmark *T*_o_ was set to match the median air temperature of the day based on weather forecasts, whereas *T*_o_ was set a few degrees below the maximum daily air temperature for the measurements in Australia in 2020 and Denmark in 2021. Hence, *T*_o_ varied between leaves both within and between all measured species but was kept constant within each leaf repeatedly measured throughout the day. The measurements were conducted using the Kok method with a relative air humidity of 35–80%, a flow rate of 300–350 μmol s^−1^, a fan speed of 10 000 rpm and a CO_2_ concentration of 410 ppm in the leaf chamber. The leaf irradiance response of *A*_net_ was measured from 100 μmol photons m^−2^ s^−1^ down to 0 μmol photons m^−2^ s^−1^ in steps of 10–12 irradiance levels. Before measuring *A*_net_ at each irradiance level, the reference and sample infrared gas analysers (IRGAs) were automatically matched, and gas exchange fluxes were given 2–5 min to stabilize. The stabilizing period for the 0 μmol photons m^−2^ s^−1^ irradiance level was increased to 10 min. Gas exchange fluxes were allowed to stabilize before initiation of the light response curve. This stabilization typically required 2–20 min.

### Calculation of leaf light and dark respiration

The Kok method was used to calculate *R*_L_ (μmol CO_2_ m^−2^ s^−1^) while *R*_D_ (μmol CO_2_ m^−2^ s^−1^) was measured following leaf dark adaptation. The apparent *R*_L_ was estimated based on the intercept of a linear regression fitted to the linear region above the Kok effect and *R*_D_ was determined directly from the CO_2_ efflux in the dark. When using the Kok method, intercellular CO_2_ concentration (*C*_i_, μmol mol^−1^) tends to increase as irradiance decreases, resulting in reduced photorespiration and increased carboxylation (Villar *et al*., 1994). As a result, the slope of *A*_net_ light response curves tends to decrease, resulting in the concurrent underestimation of *R*_L_ (Kirschbaum & Farquhar, 1987; Villar *et al*., 1994). Accordingly, rates of *R*_L_ were corrected for changes in *C*_i_ by iteratively forcing the intercept of the quantum yield of RuP_2_ regeneration, *V*_j_, against irradiance through the origin. Following this, *V*_j_ – *R*_L_ was plotted against irradiance and a linear regression was fitted to the linear region above the Kok effect and extrapolated to the *y*‐axis yielding the actual *R*_L_ (Fig. S1). *V*_j_ was calculated following Kirschbaum & Farquhar (1987):

(Eqn 1) $$V_{j}=\frac{\left(A_{\mathrm{net}}+R_{L}\right)\cdot \left(1+\frac{2\Gamma_{\text{leafT}}^{*}}{C_{i}}\right)}{1-\frac{\Gamma_{\text{leafT}}^{*}}{C_{i}}}\;$$

where Γ* (μbar) is the apparent CO_2_ compensation point in the absence of *R*_L_ (von Caemmerer & Farquhar, 1981) and *A*_net_ is the rate of net CO_2_ exchange at any given irradiance. The *C*_i_‐based Γ* at 25°C ($\Gamma_{25{}^{\circ}C}^{*}$) was assumed to be 38.6 μbar for all species, and Γ* at any given leaf temperature ($\Gamma_{\text{leafT}}^{*}$) can be calculated according to Brooks & Farquhar (1985):

(Eqn 2) $$\Gamma_{\text{leafT}}^{*}=\Gamma_{25{}^{\circ}C}^{*}+1.88\;\left(T_{\text{leaf}}\;-\;25\right)+0.036\;{\left(T_{\text{leaf}}\;-\;25\right)}^{2}.$$

The *C*_i_‐corrected *R*_L_ and the *R*_D_ estimated from the CO_2_ efflux in the dark was subsequently used to calculate the % light inhibition of *R*_D_ as 1 − (*R*_L_/*R*_D_)100. The rate of gross photosynthesis (*A*_gross_, μmol CO_2_ m^−2^ s^−1^) was calculated as *R*_L,To_ plus *A*_net_ at the 100 μmol photons m^−2^ s^−1^ irradiance level.

### Data analysis

To examine whether *R*_L_ and *R*_D_ exhibited diurnal variation measured at a constant temperature (hereafter denoted as *R*_L,To_ and *R*_D,To_), all measurements of *R*_L,To_ and *R*_D,To_ were standardized by dividing each measurement from a single leaf by the mean *R*_L,To_ or *R*_D,To_ measurement of that leaf (i.e. $R_{L,\text{To}}/\bar{R_{L,\text{To}}}$ and $R_{D,\text{To}}/\bar{R_{D,\text{To}}}$, respectively). Diurnal variations in $R_{L,\text{To}}/\bar{R_{L,\text{To}}}$ and $R_{D,\text{To}}/\bar{R_{D,\text{To}}}$, as well as the % light inhibition of *R*_D,To_ were examined using generalized additive models (GAMs) with 95% pointwise confidence intervals fitted with automated smoothness selection in the mgcv library in R v.4.1.0 (R Core Team, 2021) with Rstudio v.1.4.1717 (R Studio Team, 2021), using restricted maximum likelihood (REML) (Wood, 2017). The GAMs had the following components:

(Eqn 3) $$y_{i}=\alpha +f\left(x_{i}\right)+\varepsilon_{i}$$

where *y*_*i*_ is the observation at time *x*_*i*_, α is the intercept, *f*(*x*_*i*_) is a smooth function and *ε*_*i*_ is the residual error. This approach is nonparametric and makes no *a priori* assumption about the functional relationship between variables (Wood, 2017), allowing the depiction of the empirical trend of $R_{L,\text{To}}/\bar{R_{L,\text{To}}}$ and $R_{D,\text{To}}/\bar{R_{D,\text{To}}}$ and the % light inhibition of *R*_D,To_ over time without restrictions. The residual variation was assumed to follow a gaussian distribution, and the residual autocorrelation was modelled by a continuous time first‐order autoregressive process structure nested within each measured leaf (Pinheiro & Bates, 2000). Accordingly, GAMs by the formulation of Eqn 3 were fitted to $R_{L,\text{To}}/\bar{R_{L,\text{To}}}$ and $R_{D,\text{To}}/\bar{R_{D,\text{To}}}$ and % light inhibition of *R*_D,To_ measurements across all measurements, across measurements within climates (i.e. Australia and Denmark), within PFTs and within species. As the $R_{L,\text{To}}/\bar{R_{L,\text{To}}}$, $R_{D,\text{To}}/\bar{R_{D,\text{To}}}$ and the % light inhibition of *R*_D,To_ measurements in Denmark were represented by nine deciduous tree species and one herbaceous species, the fitting of GAMs to PFTs was restricted to Australia. Significant diurnal variations of $R_{L,\text{To}}/\bar{R_{L,\text{To}}}$, $R_{D,\text{To}}/\bar{R_{D,\text{To}}}$ and the % light inhibition of *R*_D,To_ were determined after computation of the first derivative (the slope) of the fitted GAMs with the finite differences method. The first derivatives were computed with 95% pointwise confidence intervals, and the trend was deemed significant when the derivative confidence interval was bounded away from zero at the 95% level (for more details on this method, see Curtis & Simpson, 2014). The percentage total diurnal variation was calculated from the difference between minimum and maximum predicted values of $R_{L,\text{To}}/\bar{R_{L,\text{To}}}$, $R_{D,\text{To}}/\bar{R_{D,\text{To}}}$ and the % light inhibition of *R*_D,To_, divided by the maximum GAM‐predicted values.

Model selection was used to determine whether the diurnal variation in $R_{L,\text{To}}/\bar{R_{L,\text{To}}}$, $R_{D,\text{To}}/\bar{R_{D,\text{To}}}$ and % light inhibition of *R*_D,To_ differed between climates and PFTs (Wieling, 2018). Models with the structure of Eqn 3 or with PFT or climate added as a covariate (Eqn 4) and an interaction term (Eqn 5) were fitted across climates or across all three PFTs in Australia using the following equations:

(Eqn 4) $$y_{i}=\alpha +f\left(x_{i}\right)+K_{i}+\varepsilon_{i}$$

(Eqn 5) $$y_{i}=\alpha +f\left(x_{i}\right)+K_{i}+f\left(x_{i}\right)\times k_{1,i}+\ldots +f\left(x_{i}\right)\times k_{n,i}+\varepsilon_{i}$$

where *K* is a categorical variable (i.e. climate or PFT) of *n* levels (*k*_1_ – *k*_*n*_). The variable *k*_*n*,*i*_ is 1 if an observation is from level *k*_*n*_, and otherwise 0. The models explaining the highest variance with the minimum number of variables were identified using Akaike's information criterion (AIC).

The prediction of $R_{L,\text{To}}/\bar{R_{L,\text{To}}}$, $R_{D,\text{To}}/\bar{R_{D,\text{To}}}$ and the % light inhibition of *R*_D,To_ from physiological and external environmental variables was examined using linear or logarithmic regression models with 95% pointwise confidence intervals. The ambient temperature (°C), light intensity (μmol m^−2^ s^−1^) and water vapour pressure deficit (VPD; kPa), provided by the climate station at HFE in Australia, were averaged for every hour during the time of measuring a light response curve of *A*_net_. Model assumptions of normality and homoscedasticity of residuals were assessed and verified before analysis.

To examine the potential error of assuming a constant rate of *R*_L,To_ throughout a day, the GAMs that were initially fitted to the $R_{L,\text{To}}/\bar{R_{L,\text{To}}}$ values were used to predict *R*_L,To_ throughout a day based on a hypothetical scenario, where an *R*_L,To_ measurement of 0.76 μmol CO_2_ m^−2^ s^−1^ (i.e. the mean estimated *R*_L,To_ value across all species in this study) was measured at 08:00 h. Accordingly, temporal GAMs at constant temperature were fitted to the $R_{L,\text{To}}/\bar{R_{L,\text{To}}}$ values from Australia and Denmark using Eqn 3. However, for these models we estimated 95% simultaneous confidence intervals from the multivariate normal distribution of the models that contain 95% of the posterior draws from the estimated models, according to Simpson (2018). Subsequently, these models were used to predict the *R*_L,To_ measurement of 0.76 μmol CO_2_ m^−2^ s^−1^ at other times of the day following:

(Eqn 6) $${R_{L,\text{To}}}_{i}=R_{L,\text{To}}\times \left(\frac{\alpha +f\left({\text{time}}_{i}\right)+\varepsilon_{i}}{\alpha +f\left({\text{time}}_{x}\right)+\varepsilon_{x}}\right)$$

where ${R_{L,\text{To}}}_{i}$ is the predicted rate of respiration at constant temperature at time_*i*_. *R*_L,To_ is the measured *R*_L,To_ (0.76 μmol CO_2_ m^−2^ s^−1^) at time_*x*_ (08:00 h), $\varepsilon_{x}$ is the residual error at time_*x*_ and $\varepsilon_{i}$ is the residual errors at time_*i*_. Null models (i.e. linear regression models without a slope) were fitted through the maximum, mean and minimum predicted values of the temporal GAMs for Australia and Denmark, and the predicted accumulated CO_2_ throughout a day was calculated for each model. The percentage predicted difference in accumulated CO_2_ between the maximum, mean and minimum models compared to the temporal GAMs was subsequently calculated. Eqn 6 was further merged with a Q_10_ model in order to calculate *R*_L,T_ (i.e. *R*_L_ under varying temperature conditions):

(Eqn 7) $${R_{L,T}}_{i}=\left(R_{L,\text{To}}\times \left(\frac{\alpha +f\left({\text{time}}_{i}\right)+\varepsilon_{i}}{\alpha +f\left({\text{time}}_{x}\right)+\varepsilon_{x}}\right)\right)\times 2^{\left(\frac{\left(T_{i}-T_{o}\right)}{10}\right)}$$

where 2 denotes the factor by which ${R_{L,T}}_{i}$ changes for every 10°C temperature change, ${R_{L,T}}_{i}$ is the predicted rate of respiration at temperature *T*_i_ and *T*_o_ is the temperature at the time where *R*_L,To_ was measured. Using Eqn 7, *R*_L,T_ (i.e. *R*_L_ at different temperatures) was calculated at three different temperature profiles for both climates. A parametric approach with quadratic linear regression models and 95% pointwise confidence intervals was also constructed following:

(Eqn 8) $${R_{L,T}}_{i}=\left(R_{L,\text{To}}\times \left(\frac{\alpha +\beta {\text{time}}_{i}+\beta_{1}{\text{time}}_{i}^{2}+\varepsilon_{i}}{\alpha +\beta {\text{time}}_{x}+\beta_{1}{\text{time}}_{x}^{2}+\varepsilon_{x}}\right)\right)\times 2^{\left(\frac{\left(T_{i}-T_{o}\right)}{10}\right)}$$

where α is the model intercept, β and $\beta_{1}$ are model coefficients, and ε indicates the residuals, which are assumed to follow a gaussian distribution. Using the guide in Dataset S1 and the R script (Notes S2), an *R*_L,To_ measurement from any time of the day can be used to predict *R*_L,T_ or *R*_L,To_ with either GAMs or quadratic linear regression models throughout the day depending on whether the daily temperature is constant or varying. The temperature profiles were derived from the ambient air temperature during the time of measuring the light response curves in Australia. Quadratic linear regression models were fitted through the maximum, mean and minimum temperature within 2 h intervals from 04:00 to 22:00 h from these data, yielding three different temperature profiles, respectively, which can be viewed in Fig. S2.

## Results

### Diurnal variation in leaf light and dark respiration

**Fig. 1:**
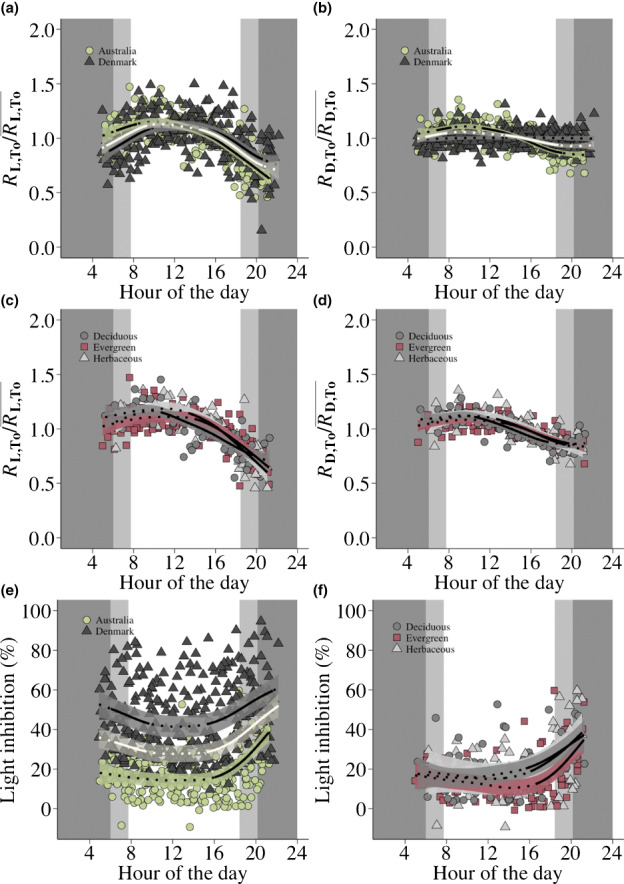
The diurnal variation of (a, c) leaf respiration in the light ($R_{L,\text{To}}/\bar{R_{L,\text{To}}}$), (b, d) leaf dark respiration ($R_{D,\text{To}}/\bar{R_{D,\text{To}}}$) and (e, f) the % light inhibition of leaf respiration (1 − (*R*_L,To_/*R*_D,To_)100). Generalized additive models (GAMs) with 95% pointwise confidence intervals are fitted across (a, b, e) measurements of 10 field‐grown plant species from Denmark (dark triangles, *n* = 295), 11 from Australia (green circles, *n* = 270) and across (c, d, f) four deciduous (dark circles, *n* = 88), four evergreen (red squares, *n* = 115) and three herbaceous (grey triangles, *n* = 65) species measured in Australia. GAMs indicated by the white lines in (a, b, e) are fitted across all measurements from Denmark and Australia (*n* = 562). Since the measurements from Denmark were represented by nine deciduous and one herbaceous species, the fitting of GAMs to PFTs was restricted to the measurements in Australia where four deciduous, four evergreen and three herbaceous species were measured. Significant variations in the diurnal variation of $R_{L,\text{To}}/\bar{R_{L,\text{To}}}$, $R_{D,\text{To}}/\bar{R_{D,\text{To}}}$ and the % light inhibition of leaf respiration are indicated by the solid portions of the fitted GAMs while dotted portions illustrate nonsignificant variations. Dark shaded areas illustrate night‐time shared for all days of measuring and light shaded areas illustrate the variation in time of sunrise and sunset between the days of measuring. The studied species are detailed in Supporting Information Table S1.

When standardizing *R*_L,To_ and *R*_D,To_ at the individual leaf level, $R_{L,\text{To}}/\bar{R_{L,\text{To}}}$ and $R_{D,\text{To}}/\bar{R_{D,\text{To}}}$ increased significantly from sunrise until morning or early midday, stabilized and then decreased significantly from late midday until sunset (Figs 1a,b, S3). Although $R_{L,\text{To}}/\bar{R_{L,\text{To}}}$ and $R_{D,\text{To}}/\bar{R_{D,\text{To}}}$ showed significant variations at approximately the same time of day, the total diurnal variation in the fitted models was larger for $R_{L,\text{To}}/\bar{R_{L,\text{To}}}$ compared to $R_{D,\text{To}}/\bar{R_{D,\text{To}}}$ (38% and 12% change, respectively, Table S2). In addition, the diurnal patterns showed no linear association with the various preset constant leaf measurement temperatures (*r* ^2^ = 1.234667 × 10^−29^, *P* > 0.05 and *r* ^2^ = 3.505181 × 10^−30^, *P* > 0.05, for $R_{L,\text{To}}/\bar{R_{L,\text{To}}}$ and $R_{D,\text{To}}/\bar{R_{D,\text{To}}}$, respectively) (Fig. S4), showing that the diurnal patterns were persistent across a wide range of leaf temperatures.

The diurnal variation of $R_{L,\text{To}}/\bar{R_{L,\text{To}}}$ and $R_{D,\text{To}}/\bar{R_{D,\text{To}}}$ persisted when analysing measurements within climates, and this pattern differed between the climates for both $R_{L,\text{To}}/\bar{R_{L,\text{To}}}$ and $R_{D,\text{To}}/\bar{R_{D,\text{To}}}$ (Fig. 1a,b; Table S3). For Australia, $R_{L,\text{To}}/\bar{R_{L,\text{To}}}$ increased significantly from sunrise until morning, stabilized and then decreased significantly from early midday until sunset (Fig. 1a). By contrast, $R_{D,\text{To}}/\bar{R_{D,\text{To}}}$ was stable from sunrise until early midday, and then decreased significantly until sunset (Fig. 1b). In addition, $R_{L,\text{To}}/\bar{R_{L,\text{To}}}$ exhibited a larger total variation in the fitted model compared to $R_{D,\text{To}}/\bar{R_{D,\text{To}}}$ (45% and 24% change, respectively, Table S2). In Denmark, $R_{L,\text{To}}/\bar{R_{L,\text{To}}}$ increased significantly from sunrise until early midday, stabilized and then decreased significantly from late midday until sunset (Fig. 1a) and the total variation in the fitted model was comparable to that of $R_{L,\text{To}}/\bar{R_{L,\text{To}}}$ in Australia (33% change, Table S2). By contrast, $R_{D,\text{To}}/\bar{R_{D,\text{To}}}$ did not exhibit a significant diurnal variation in Denmark, but remained stable throughout the day with only minimal total variation in the fitted model (Fig. 1b; Table S2). All measured species in Australia exhibited significant diurnal variations in $R_{L,\text{To}}/\bar{R_{L,\text{To}}}$ and $R_{D,\text{To}}/\bar{R_{D,\text{To}}}$ (Figs S5–S7). In Denmark, eight out of 10 species exhibited significant diurnal variations in $R_{L,\text{To}}/\bar{R_{L,\text{To}}}$, while five exhibited significant diurnal variations in $R_{D,\text{To}}/\bar{R_{D,\text{To}}}$ (Figs S8–S10).

The PFTs measured in Australia showed similar diurnal variations in both $R_{L,\text{To}}/\bar{R_{L,\text{To}}}$ and $R_{D,\text{To}}/\bar{R_{D,\text{To}}}$ (Fig. 1c,d; Table S3). For each PFT, $R_{L,\text{To}}/\bar{R_{L,\text{To}}}$ and $R_{D,\text{To}}/\bar{R_{D,\text{To}}}$ were stable from sunrise until early or late midday, and decreased significantly until late evening or sunset (Fig. 1c,d). The total variation in the fitted models for $R_{L,\text{To}}/\bar{R_{L,\text{To}}}$ and $R_{D,\text{To}}/\bar{R_{D,\text{To}}}$ was of similar magnitude between the PFTs (Deciduous: 40% and 21% change, Evergreen: 42% and 23% change, Herbaceous: 49% and 28% change, respectively, Table S2), although the total variation of $R_{L,\text{To}}/\bar{R_{L,\text{To}}}$ was slightly larger than that of $R_{D,\text{To}}/\bar{R_{D,\text{To}}}$ for all PFTs.

### Diurnal variation in the light inhibition of *R*_D_ _,To_

Across climates, the light inhibition of *R*_D,To_ decreased significantly from sunrise until morning, stabilized and then increased significantly from late midday until sunset (Fig. 1e). The variation in the inhibition values ranged between −9% and 95% with a mean inhibition of 32% (Table S4) and the total variation in the fitted model was 47% (Table S2), emphasizing that *R*_L,To_ and *R*_D,To_ exhibited different diurnal patterns.

The diurnal variation of the light inhibition of *R*_D,To_ differed between the climates (Fig. 1e; Table S3). For Denmark, the light inhibition of *R*_D,To_ decreased significantly from sunrise until morning, stabilized and then increased significantly from late midday until sunset (Fig. 1e). For Australia, the light inhibition of *R*_D,To_ was stable from sunrise until late midday, and then increased significantly from late midday until sunset (Fig. 1e). The total variation in the fitted model for Denmark was 32% and the mean inhibition was 45% (Tables S2, S4) while the total variation in the fitted model for Australia was 63% and the mean inhibition was 18% (Tables S2, S4, respectively). In addition, the variation in the inhibition values was larger for Denmark than for Australia (Fig. 1e; Table S4).

The diurnal variation of the light inhibition of *R*_D,To_ differed between the PFTs measured in Australia (Fig. 1f; Table S3). For each PFT, the light inhibition of *R*_D,To_ was stable from sunrise until late midday or evening, and then increased significantly until sunset (Fig. 1f). However, the evergreen PFT showed a more abrupt increase in the evening compared to the other PFTs. In addition, the total variation in the fitted models was 58%, 70% and 61% for the deciduous, evergreen and herbaceous PFT, respectively (Table S2), and the mean and range in inhibition were similar among the three (Table S4).

### Importance of photosynthetic rate and stomatal conductance

**Fig. 2:**
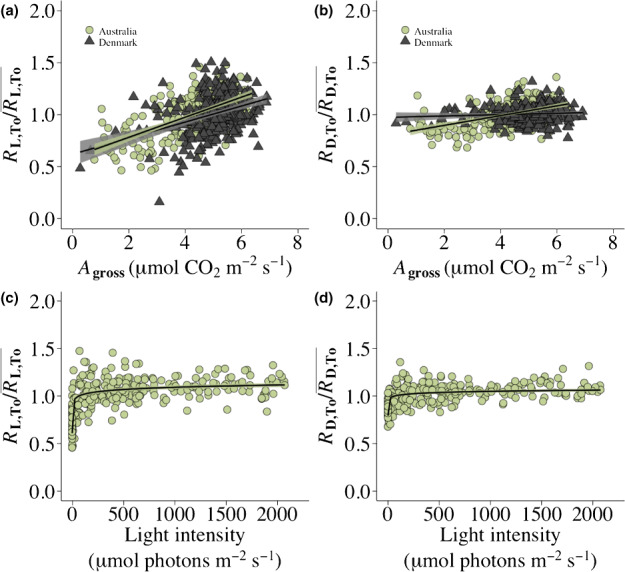
Leaf respiration (a) in the light ($R_{L,\text{To}}/\bar{R_{L,\text{To}}}$) and (b) leaf dark respiration ($R_{D,\text{To}}/\bar{R_{D,\text{To}}}$) measurements conducted in Australia (green circles, *n* = 268) and Denmark (dark triangles, *n* = 294) plotted against *A*_gross_ (μmol CO_2_ m^−2^ s^−1^) at the 100 μmol photons m^−2^ s^−1^ irradiance level. Leaf respiration (c) in the light ($R_{L,\text{To}}/\bar{R_{L,\text{To}}}$) and (d) leaf dark respiration ($R_{D,\text{To}}/\bar{R_{D,\text{To}}}$) measurements conducted in Australia plotted against the recorded mean ambient light intensity (μmol photons m^−2^ s^−1^) during the time of measuring the light response curves. Linear regression models with 95% pointwise confidence intervals are fitted to the (a) $R_{L,\text{To}}/\bar{R_{L,\text{To}}}$ ($y_{i}=0.581728+0.096533A_{{\text{gross}}_{i}}+\varepsilon_{i}$, *r* ^2^ = 0.39, *P* < 0.05 and $y_{i}=0.61213+0.07729A_{{\text{gross}}_{i}}+\varepsilon_{i}$, *r* ^2^ = 0.14, *P* < 0.05), (b) $R_{D,\text{To}}/\bar{R_{D,\text{To}}}$ ($y_{i}=0.796791+0.046899A_{{\text{gross}}_{i}}+\varepsilon_{i}$, *r* ^2^ = 0.24, *P* < 0.05 and $y_{i}=0.967332+0.006510A_{{\text{gross}}_{i}}+\varepsilon_{i}$, *r* ^2^ = 0.006, *P* > 0.05) measurements in Australia and Denmark, respectively. Logarithmic regression models with 95% pointwise confidence intervals are fitted to the (c) $R_{L,\text{To}}/\bar{R_{L,\text{To}}}$ ($y_{i}=0.858178+0.033641\log_{e}\left({\text{light}}_{i}\right)+\varepsilon_{i}$, *r* ^2^ = 0.49, *P* < 0.05) and (d) $R_{D,\text{To}}/\bar{R_{D,\text{To}}}$ ($y_{i}=0.924902+0.017814\log_{e}\left({\text{light}}_{i}\right)+\varepsilon_{i}$, *r* ^2^ = 0.36, *P* < 0.05) measurements in Australia. The studied species are detailed in Supporting Information Table S1.

There was a significant positive linear relationship between $R_{L,\text{To}}/\bar{R_{L,\text{To}}}$ and *A*_gross_ at the 100 μmol photons m^−2^ s^−1^ irradiance level for the measurements collected in Australia (*r* ^2^ = 0.39, *P* < 0.05) and for the measurements collected in Denmark (*r* ^2^ = 0.14, *P* < 0.05) (Fig. 2a). *A*_gross_ showed a significant positive linear relationship with the $R_{D,\text{To}}/\bar{R_{D,\text{To}}}$ measurements from Australia (*r* ^2^ = 0.24, *P* < 0.05) while there was no significant association between *A*_gross_ and the $R_{D,\text{To}}/\bar{R_{D,\text{To}}}$ measurements from Denmark (*r* ^2^ = 0.006, *P* > 0.05) (Fig. 2b). *A*_gross_ showed a significant positive logarithmic relationship with *g*_sw_ for the measurements collected in Australia (*r* ^2^ = 0.71, *P* < 0.05) and for the measurements in Denmark (*r* ^2^ = 0.35, *P* < 0.05) while *g*_sw_ was in general higher in Denmark (Fig. S11). The light inhibition of *R*_D,To_ showed a negative linear association with *A*_gross_ in Australia (*r* ^2^ = 0.30, *P* < 0.05) and in Denmark (Fig. S12a) (*r* ^2^ = 0.04, *P* < 0.05).

### Influence of external environmental factors on the diurnal patterns of leaf light and dark respiration

There was a significant positive logarithmic relationship between the ambient light intensity and the $R_{L,\text{To}}/\bar{R_{L,\text{To}}}$ (*r* ^2^ = 0.49, *P* < 0.05) and $R_{D,\text{To}}/\bar{R_{D,\text{To}}}$ (*r* ^2^ = 0.36, *P* < 0.05) measurements in Australia, such that when the ambient light intensity exceeded *c*. 100 μmol photons m^−2^ s^−1^ there was no apparent influence of the ambient light intensity on the $R_{L,\text{To}}/\bar{R_{L,\text{To}}}$ and $R_{D,\text{To}}/\bar{R_{D,\text{To}}}$ measurements (Fig. 2c,d). In a similar manner, the light inhibition of *R*_D,To_ showed a logarithmic relationship with ambient light intensity although here the relationship was negative (*r* ^2^ = 0.21, *P* < 0.05). In addition, ambient temperature and VPD displayed no relationship with the $R_{L,\text{To}}/\bar{R_{L,\text{To}}}$ and $R_{D,\text{To}}/\bar{R_{D,\text{To}}}$ measurements (Figs S13a,b, S14a,b).

### Error of assuming a constant daytime respiration at constant temperature for modelling integrated respiratory carbon flux in leaves

**Fig. 3:**
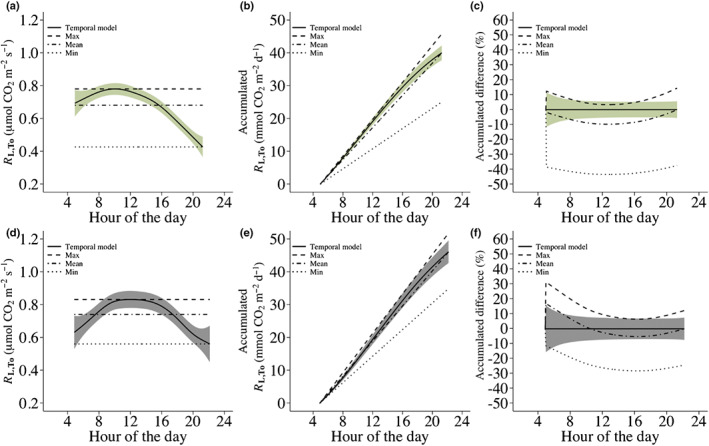
Temporal generalized additive models (GAMs) of respiration in the light at constant temperature (*R*_L,To_) for Australia (a) and Denmark (d), where a hypothetical *R*_L,To_ value of 0.76 μmol CO_2_ m^−2^ s^−1^ (i.e. the mean estimated *R*_L,To_ value across all species in this study) measured at 08:00 h was used to predict *R*_L,To_ values throughout the day at a constant temperature (solid line) with shaded 95% simultaneous confidence bands. The maximum, mean and minimum models (dashed, dot dashed and dotted lines, respectively) are fitted through the maximum, mean and minimum predicted values of the temporal GAMs, respectively, and assume a constant rate of *R*_L,To_ throughout the day. The predictions were derived by fitting GAMs to the $R_{L,\text{To}}/\bar{R_{L,\text{To}}}$ measurements from Australia and Denmark. Subsequently, the hypothetical *R*_L,To_ value measured at 08:00 h was used to predict *R*_L,To_ throughout the day from the fitted GAM from Australia as: ${R_{L,\text{To}}}_{i}$ = 0.76((0.998728 + *f*(time_*i*_) + $\varepsilon_{i}$)/(0.998728 + *f*(time_*x*_) + $\varepsilon_{x}$)) and from the fitted GAM from Denmark as: ${R_{L,\text{To}}}_{i}$ = 0.76((1.00074 + *f*(time_*i*_) + $\varepsilon_{i}$)/(1.00074 + *f*(time_*x*_) + $\varepsilon_{x}$)), where 0.76 is the *R*_L,To_ value measured at 08:00 h (time_*x*_), $\varepsilon_{x}$ is the estimated residual error at 08:00 h, ${R_{L,\text{To}}}_{i}$ is the predicted rate of respiration at time points time_*i*_ and $\varepsilon_{i}$ is the residual error at time_*i*_. (b, e) Daily accumulated CO_2_ efflux predicted by the temporal, max, mean and min models for Australia and Denmark, respectively. (c, f) Percentage difference between accumulated CO_2_ predicted by the maximum, mean and minimum models and the temporal models for Australia and Denmark, respectively.

A hypothetical *R*_L,To_ value of 0.76 μmol CO_2_ m^−2^ s^−1^ (the mean *R*_L,To_ of this study) measured at 08:00 h was used to predict *R*_L,To_ throughout the day at a constant temperature for both Australia and Denmark (i.e. temporal models), as described in Eqn 6 (Fig. 3a,d). In addition, models assuming a constant *R*_L,To_ throughout the day were fitted to the maximum, mean and minimum *R*_L,To_ values predicted by the temporal models (Fig. 3a,d). For Australia, the predicted daily accumulated CO_2_ efflux using the temporal, maximum, mean and minimum models was 40, 46, 40 and 25 mmol m^−2^ d^−1^, respectively (Fig. 3b). For Denmark, the predicted daily accumulated CO_2_ efflux was 46, 52, 46 and 35 mmol m^−2^ d^−1^, respectively (Fig. 3e). For Australia, the percentage difference in accumulated CO_2_ between the maximum, mean and minimum models and the temporal model was 14%, −0.09% and −37%, respectively (Fig. 3c), while for Denmark, the percentage difference was 12%, −0.1% and −24%, respectively (Fig. 3f). Hence, the magnitude and direction of the error resulting from assuming a constant rate of *R*_L,To_ throughout a day is highly dependent on the time of measurement.

### Daytime respiration at varying and constant temperature

**Fig. 4:**
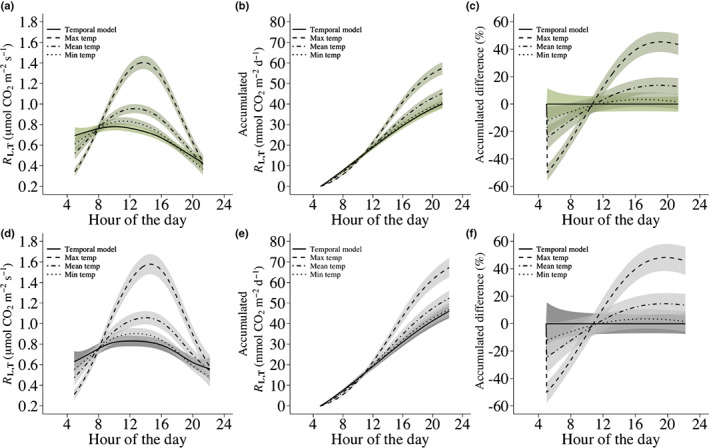
Temporal generalized additive models (GAMs) of respiration in the light at constant temperature (*R*_L,To_) (solid line) and maximum, mean and minimum temperature variation models of respiration in the light at varying temperature (*R*_L,T_) (dashed, dot dashed and dotted lines, respectively) with shaded 95% simultaneous confidence bands for Australia (a) and Denmark (d). For the temporal GAMs, a hypothetical *R*_L,To_ value of 0.76 μmol CO_2_ m^−2^ s^−1^ (i.e. the mean estimated *R*_L,To_ value across all species in this study) measured at 08:00 h was used to predict *R*_L,To_ values throughout the day at a constant temperature. The maximum, mean and minimum temperature variation models are based on three temperature profiles and predict *R*_L,T_. The predictions of *R*_L,T_ were derived by fitting GAMs to the $R_{L,\text{To}}/\bar{R_{L,\text{To}}}$ measurements from Australia and Denmark. Subsequently, the hypothetical *R*_L,To_ value measured at 08:00 h was used to predict *R*_L,T_ throughout the day at different temperatures from the fitted GAM from Australia as: ${R_{L,T}}_{i}=\left(0.76\left(\left(0.998728+f\left({\text{time}}_{i}\right)+\varepsilon_{i}\right)/\left(0.998728+f\left({\text{time}}_{x}\right)+\varepsilon_{x}\right)\right)\right)2^{\left(T_{i}–T_{o}/10\right)}$ and from the fitted GAM from Denmark as: ${R_{L,T}}_{i}=\left(0.76\left(\left(1.00074+f\left({\text{time}}_{i}\right)+\varepsilon_{i}\right)/\left(1.00074+f\left({\text{time}}_{x}\right)+\varepsilon_{x}\right)\right)\right)2^{\left(T_{i}–T_{o}/10\right)}$, where 0.76 is the *R*_L,To_ value measured at 08:00 h (time_*x*_), $\varepsilon_{x}$ is the estimated residual error at 08:00 h, ${R_{L,T}}_{i}$ is the predicted rate of respiration at temperatures *T*_*i*_ and time point time_*i*_, and $\varepsilon_{i}$ is the residual errors at time_*i*_. *T*_o_ is the temperature at the time where the *R*_L,To_ value of 0.76 was measured and 2 denotes the factor by which ${R_{L,T}}_{i}$ changes for every 10°C temperature change. The temperature profiles were derived from the measured ambient temperature during the time of measuring the light response curves in Australia and can be viewed in Supporting Information Fig. S2. (b, e) Daily accumulated CO_2_ efflux predicted by the temporal, maximum temperature, mean temperature and minimum temperature variation models for Australia and Denmark, respectively. (c, f) Percentage difference between accumulated CO_2_ predicted by the maximum temperature, mean temperature and minimum temperature variation models and the temporal models for Australia and Denmark, respectively.

The predicted diurnal variation of *R*_L,To_ using the temporal GAMs was compared to the predicted diurnal variation of *R*_L,T_ had the leaf temperature varied with a maximum, mean and minimum air temperature profile (Fig. 4a,d). For Australia, the predicted accumulated CO_2_ for the temporal model (at constant temperature) and the maximum, mean and minimum temperature profile models was 40, 57, 45 and 41 mmol m^−2^ d^−1^, respectively (Fig. 4b) while for Denmark, the predicted accumulated CO_2_ was 46, 67, 52 and 47 mmol m^−2^ d^−1^, respectively (Fig. 4e). For Australia, the percentage difference in accumulated CO_2_ between the maximum, mean and minimum temperature profile models and the temporal model was 44%, 13% and 2%, respectively (Fig. 4c). For Denmark, this difference was 46%, 14% and 2%, respectively (Fig. 4f). Figures for the same analysis using quadratic linear regression models can be found in Fig. S15.

## Discussion

### *R*_L_ and *R*_D_ measured with the Kok method varies over the course of the day at constant measuring temperature

This study shows that leaf daytime respiration exhibits a consistent temporal pattern throughout the day when measured at constant temperature with the Kok method across 21 different plant species, two different climates and three different PFTs, emphasizing the generality of the phenomenon. Leaf daytime respiration is thus clearly driven by time of day, when estimated as both *R*_L_ and *R*_D_. Assuming a constant rate of *R*_L_ at constant temperature can result in the over‐ or underestimation of the accumulated daily *R*_L,To_ by 14% and −37% or 12% and −24% on a diurnal scale as demonstrated for Australia and Denmark, respectively (Fig. 3c,f). This variation in *R*_L_ is of similar magnitude to that of the variation in *R*_L_ attributed to other studied factors (e.g. CO_2_: 32% (Ayub *et al*., 2014), nutrient availability: 29% (Crous *et al*., 2017a), drought: *c*. 52–60% (Ayub *et al*., 2011; Crous *et al*., 2012), canopy height: 52% (Weerasinghe *et al*., 2014), seasonality: 32% (Crous *et al*., 2017b) and temperature: −15 to 90% per every 10°C temperature increase (Atkin *et al*., 2005)). Errors rising when comparing rates of respiration sampled at different times of the day could potentially bias conclusions on the influence of such effects. Hence, time of day should be accounted for when estimating the response of *R*_L_ and *R*_D_ to variation in other factors (e.g. temperature and species), or when pooling data across studies even when measurements are performed with the same temperature. Effects of time of day may result in variations of *R*_L,To_ that in turn affect the calculation of photosynthetic parameters important for photosynthetic modelling. These could include estimates of maximum carboxylase (*V*_cmax_) rates estimated with the one‐point *A*_sat_ method, which has been shown to be sensitive to the chosen *R*_L_ (De Kauwe *et al*., 2016) and thereby affected by time of day.

### Light inhibition of *R*_D_ _,To_ exhibited significant diurnal variations

The light inhibition of *R*_D,To_ exhibited significant diurnal variations with inhibition values ranging from −9% to 95% and with a mean of 32%, reflecting that the diurnal pattern differed between $R_{L,\text{To}}/\bar{R_{L,\text{To}}}$ and $R_{D,\text{To}}/\bar{R_{D,\text{To}}}$. This indicates that *R*_L_ and *R*_D_ are regulated by different processes, as supported by ample biochemical evidence (Hurry *et al*., 2005; Tcherkez *et al*., 2010, 2012a, 2017b) and by studies showing that the temperature sensitivity of *R*_L_ may differ from that of *R*_D_ (Atkin *et al*., 2005; Zaragoza‐Castells *et al*., 2007; McLaughlin *et al*., 2014; Kroner & Way, 2016; Crous *et al*., 2017b). The light inhibition of *R*_D_ has been shown to vary between 0 and 100% when estimated with the Kok and Laisk method (Atkin *et al*., 2006; Zaragoza‐Castells *et al*., 2007; Crous *et al*., 2012; Heskel *et al*., 2013) and sometimes *R*_L_ even exceeds *R*_D_ (Zaragoza‐Castells *et al*., 2007; Atkin *et al*., 2013; Crous *et al*., 2017a) as demonstrated in this study as well for three measurements. Given this variability, approaches where *R*_L_ is calculated from measurements of *R*_D_, by assuming *R*_L_ constitutes a fixed fraction of *R*_D_ (e.g the JULES model), may be erroneous. The light inhibition of *R*_D,To_ showed only a weak association with the external light intensity above *c*. 100 μmol photons m^−2^ s^−1^ in Australia and decreased in a linear fashion with increasing *A*_gross_ for both Australia and Denmark, as shown previously (Atkin *et al*., 2013). However, the cause of the large difference in variability of the light inhibition of *R*_D,To_ between climates, with much higher inhibition values in Denmark, is unknown. Eight out of 10 species were measured in autumn in Denmark where inhibition values have been shown to increase (Heskel *et al*., 2014) whereas lower inhibition values have been reported earlier in the growing season (Crous *et al*., 2012; Heskel *et al*., 2014). Seasonal differences in light inhibition may contribute to the observed variability because the degree of inhibition has been shown to decrease under environmental conditions that increase the demand for energy and C skeletons, such as elevated CO_2_ (Wang *et al*., 2001; Shapiro *et al*., 2004) and increased soil nutrient availability (Heskel *et al*., 2012).

### Mechanisms related to the diurnal variation of respiration in the light at constant measuring temperature

Many processes can affect respiratory CO_2_ effluxes of leaves in the light (Tcherkez *et al*., 2017b). These include the oxidative pentose phosphate pathway (Buckley & Adams, 2011; Shameer *et al*., 2019; Xu *et al*., 2021), photorespiration (Igamberdiev *et al*., 2001; Tcherkez *et al*., 2005, 2008, 2012a), the continued utilization of stored organic acids (Gauthier *et al*., 2010), the activity of the pyruvate dehydrogenase complex (Budde & Randall, 1990; Gemel & Randall, 1992), the activity of the malic enzyme (Gauthier *et al*., 2020) and NAD(P)H : NAD(P) ratios (Igamberdiev & Gardeström, 2003). Some CO_2_ fluxes may even originate from nonleaf sources that are transported through the vascular tissues to leaves and subsequently released (Stutz *et al*., 2017; Stutz & Hanson, 2019). It is therefore paramount to study how such processes are temporally coregulated and affect CO_2_ effluxes in the light.

The diurnal variation of $R_{L,\text{To}}/\bar{R_{L,\text{To}}}$ and $R_{D,\text{To}}/\bar{R_{D,\text{To}}}$ showed a notable consistency throughout the study period. The wide range of measurement temperatures between the days of measuring showed no association with $R_{L,\text{To}}/\bar{R_{L,\text{To}}}$ and $R_{D,\text{To}}/\bar{R_{D,\text{To}}}$, and the ambient temperature, VPD and external light intensity above *c*. 100 μmol photons m^−2^ s^−1^ showed only weak associations with $R_{L,\text{To}}/\bar{R_{L,\text{To}}}$ and $R_{D,\text{To}}/\bar{R_{D,\text{To}}}$. This does not explicitly imply that variations in these factors were unimportant, but given the consistency of the diurnal patterns, it should be considered whether circadian regulation could play a role. *R*_D_, light‐enhanced dark respiration (LEDR) (Gessler *et al*., 2017), *g*_s_, *A*_net_ (Hennessey *et al*., 1993; Dodd *et al*., 2014) and indirectly *R*_L_ (Doughty *et al*., 2006; Resco de Dios *et al*., 2012) have been shown to be under circadian control, and further research is needed to shed light on the importance of circadian regulation in leaf daytime respiration.

Previous work has shown that photosynthesis regulates *R*_L_ through ATP utilization in sucrose synthesis, redox maintenance and substrate supply (Krömer *et al*., 1988; Raghavendra *et al*., 1994; Krömer, 1995; Hoefnagel *et al*., 1998), and a coupling of *R*_L_ to the rate of photosynthesis would explain the association between $R_{L,\text{To}}/\bar{R_{L,\text{To}}}$ and *A*_gross_ observed in both Australia and Denmark. The rate of photosynthesis may additionally have influenced the regulation of $R_{D,\text{To}}/\bar{R_{D,\text{To}}}$ given the effect of accumulated net CO_2_ assimilation on *R*_D_ due to LEDR (Azcón‐Bieto & Osmond, 1983; Hymus *et al*., 2005; Barbour *et al*., 2011). The fact that $R_{L,\text{To}}/\bar{R_{L,\text{To}}}$ and $R_{D,\text{To}}/\bar{R_{D,\text{To}}}$ exhibited different diurnal patterns within climates may be related to the differential regulation of *R*_L_ and *R*_D_ (Hurry *et al*., 2005; Tcherkez *et al*., 2010, 2017b), and to the participation of photosynthesis in the regulatory process because the availability of substrates for *R*_L_ and *R*_D_ are influenced by time of day (Pärnik *et al*., 2002, 2007; Nogués *et al*., 2004; Pärnik & Keerberg, 2007; Florez‐Sarasa *et al*., 2012; Griffin & Turnbull, 2012). In this study, stomatal conductance showed a strong association with *A*_gross_ in Australia. This could indicate an indirect stomatal regulation of $R_{L,\text{To}}/\bar{R_{L,\text{To}}}$ and $R_{D,\text{To}}/\bar{R_{D,\text{To}}}$ because of the positive linear relationship between *A*_gross_ and the two. In Denmark, stomatal conductance showed a weak association with *A*_gross_ compared to Australia, and *A*_gross_ only showed a weak positive linear relationship with $R_{L,\text{To}}/\bar{R_{L,\text{To}}}$ and none with $R_{D,\text{To}}/\bar{R_{D,\text{To}}}$. This suggests that the difference in the diurnal patterns of $R_{L,\text{To}}/\bar{R_{L,\text{To}}}$ and $R_{D,\text{To}}/\bar{R_{D,\text{To}}}$ between Australia and Denmark were an indirect result of stomatal regulation through differences in environmental factors between the climates. The fact that the PFTs measured in Australia exhibited similar diurnal patterns in $R_{L,\text{To}}/\bar{R_{L,\text{To}}}$ and $R_{D,\text{To}}/\bar{R_{D,\text{To}}}$ supports this. In addition, the fact that different species were measured in Australia and Denmark also provides a plausible explanation for this phenomenon.

In this study, increased *C*_i_ at decreasing irradiance levels was corrected using Eqn 1, but this approach assumes infinite *g*_m_ and therefore *C*_i_ = *C*_c_, which is known to be unlikely under some conditions (Harley *et al*., 1992; Flexas *et al*., 2007; Yin & Struik, 2009). In this study, diurnal changes in VPD were presumably much higher in Australia compared to Denmark, thereby making it more likely that *g*_m_ and *g*_s_ would exhibit asynchronous diurnal patterns in Australia. As a result, the *C*_i_ = *C*_c_ assumption would be erroneous, which could explain some of the diurnal variation in $R_{L,\text{To}}/\bar{R_{L,\text{To}}}$ and the light inhibition of *R*_D,To_ observed in Australia. It thus seems unlikely that the diurnal variation of $R_{L,\text{To}}/\bar{R_{L,\text{To}}}$ and the light inhibition of *R*_D,To_ is solely a result of a differential regulation of *g*_m_ and *g*_s_.

### Accounting for effects of time of day when measuring *R*_L_ using the Kok method

This study shows that time of day can have considerable effects on estimates of *R*_L_ conducted in the field with the Kok method. Comparisons of *R*_L_ between leaves should only be made at the same time of the day with the recognition that leaves may have been exposed to different environmental conditions before being measured. For computing daily averages, measurements should cover the entire photoperiod for a given leaf to yield precise estimates of daily accumulated CO_2_ effluxes. Measuring throughout the entire photoperiod is difficult experimentally especially because measurements need to be at the leaf level, which might not be possible in all experimental designs. This study offers a parametric and nonparametric approach whereby an *R*_L,To_ measurement for a given leaf can be predicted throughout the day at other temperatures using the supplementary Excel spreadsheet and R script (Dataset S1; Notes S2) based on Eqns 7 and 8, respectively. The approach can easily be implemented to other models (e.g. *V*_cmax_) by inserting the estimated model coefficients from Fig. S15 into Eqn 8. The method assumes *R*_L_ follows a distinct relative diurnal pattern (i.e. the predictions may vary in absolute values but not in per cent) regardless of environmental factors other than temperature as well as species and PFTs. Predictions should only be based on *R*_L_ measurements derived with the Kok method.

### Conclusion

This study demonstrates that *R*_L_, *R*_D_ and the light inhibition of *R*_D_ exhibit significant diurnal variations when measured with a constant temperature in the field with the Kok method. The diurnal pattern of *R*_L_ and *R*_D_ differed in trajectory and magnitude between climates but not between PFTs, while it differed both between climates and in trajectory between PFTs for the light inhibition of *R*_D_. The results emphasize that time of day should be accounted for in studies seeking to estimate the response of leaf daytime respiration to variation in other factors (e.g. temperature and species), or when deriving inference across studies. The results highlight the dynamic nature of leaf daytime respiration that are driven by factors other than the measuring temperature and that *R*_L_ and *R*_D_ exhibit distinct diurnal patterns. Temporal variation in the regulatory relationship between physiological mechanisms and leaf respiration needs further attention to unveil the drivers of leaf respiration.

## Author contributions

AHF, DB, JY, KLG, MP and MGT planned and designed the research. AHF performed experiments, conducted field work and analysed the data. AHF and DB drafted the manuscript. All authors contributed to and revised the manuscript.

## Supporting information


**Dataset S1** Excel spreadsheet containing the data used in this study including a guide on how to calculate *R*_L,T_ throughout the day by taking into account the temporal variation in *R*_L,To_ using the supplemented R script.

**Fig. S1** Example of a light response curve of measured and *C*_i_‐corrected leaf net CO_2_ exchange measurements plotted against photosynthetically active radiation.
**Fig. S2** Mean ambient temperature (°C) during the time of measuring the light response curves in Australia with fitted quadratic linear regression models depicting the maximum, mean and minimum temperature profiles.
**Fig. S3** Diurnal variation of leaf respiration in the light (*R*_L,To_) and leaf dark respiration (*R*_D,To_) from Australia and Denmark.
**Fig. S4** Leaf respiration in the light ($R_{L,\text{To}}/\bar{R_{L,\text{To}}}$) and leaf dark respiration ($R_{D,\text{To}}/\bar{R_{D,\text{To}}}$) measurements across Australia and Denmark plotted against the preset leaf measuring temperature during the time of measuring the light response curves of each leaf.
**Fig. S5** Diurnal variation of leaf respiration in the light, $R_{L,\text{To}}/\bar{R_{L,\text{To}}}$, and leaf dark respiration, $R_{D,\text{To}}/\bar{R_{D,\text{To}}}$, of *Solanum nigrum*, *Eucalyptus saligna*, *Eucalyptus tereticornis* and *Eucalyptus parramattensis* from Australia with fitted generalized additive models.
**Fig. S6** Diurnal variation of leaf respiration in the light, $R_{L,\text{To}}/\bar{R_{L,\text{To}}}$, and leaf dark respiration, $R_{D,\text{To}}/\bar{R_{D,\text{To}}}$, of *Carya illinoinensis*, *Dichondra repens*, *Eucalyptus camaldulensis* and *Araujia sericifera* from Australia with fitted generalized additive models.
**Fig. S7** Diurnal variation of leaf respiration in the light, $R_{L,\text{To}}/\bar{R_{L,\text{To}}}$, and leaf dark respiration, $R_{D,\text{To}}/\bar{R_{D,\text{To}}}$, of *Malus domestica*, *Liriodendron tulipifera* and *Platanus acerifolia* from Australia with fitted generalized additive models.
**Fig. S8** Diurnal variation of leaf respiration in the light, $R_{L,\text{To}}/\bar{R_{L,\text{To}}}$, and leaf dark respiration, $R_{D,\text{To}}/\bar{R_{D,\text{To}}}$,of *Betula pendula*, *Quercus robur*, *Fraxinus excelsior* and *Salix cinerea* from Denmark with fitted generalized additive models.
**Fig. S9** Diurnal variation of leaf respiration in the light, $R_{L,\text{To}}/\bar{R_{L,\text{To}}}$, and leaf dark respiration, $R_{D,\text{To}}/\bar{R_{D,\text{To}}}$, of *Alnus viridis*, *Alnus glutinosa*, *Helianthus annus* and *Corylus avellana* from Denmark with fitted generalized additive models.
**Fig. S10** Diurnal variation of leaf respiration in the light, $R_{L,\text{To}}/\bar{R_{L,\text{To}}}$, and leaf dark respiration, $R_{D,\text{To}}/\bar{R_{D,\text{To}}}$, of *Cornus sanguinea* and *Malus sylvestris* from Denmark with fitted generalized additive models.
**Fig. S11** *A*_gross_ at 100 μmol photons m^−2^ s^−1^ irradiance conducted in Australia and Denmark plotted against *g*_sw_ at 100 μmol photons m^−2^ s^−1^ irradiance.
**Fig. S12** Light inhibition of respiration measurements conducted in Australia and Denmark plotted against *A*_gross_ at 100 μmol photons m^−2^ s^−1^ irradiance, and against the ambient light intensity.
**Fig. S13** Leaf respiration in the light ($R_{L,\text{To}}/\bar{R_{L,\text{To}}}$) and leaf dark respiration ($R_{D,\text{To}}/\bar{R_{D,\text{To}}}$) measurements conducted in Australia plotted against the recorded mean ambient temperature (°C) during the time of measuring the light response curves.
**Fig. S14** Leaf respiration in the light ($R_{L,\text{To}}/\bar{R_{L,\text{To}}}$) and leaf dark respiration ($R_{D,\text{To}}/\bar{R_{D,\text{To}}}$) measurements conducted in Australia plotted against the recorded mean ambient vapour pressure deficit (kPa) during the time of measuring the light response curves.
**Fig. S15** Temporal quadratic linear regression models of respiration in the light at constant temperature (*R*_L,To_) and maximum, mean and minimum temperature variation models of respiration in the light at varying temperature (*R*_L,T_) for Australia and Denmark, respectively.
**Notes S1** Supplementary site description: precipitation and temperature data from 2011 to 2021 and during the time of data collection for the region covering the Danish study sites and the study site in Australia.

**Notes S2** R code to calculate temporal patterns of *R*_L,T_ while accounting for temporal variations in *R*_L,To_.
**Table S1** Measured species in Denmark and Australia with individuals from three different plant functional types (PFTs) (deciduous, herbaceous and evergreen).
**Table S2** Total diurnal variation (%) of generalized additive models fitted to $R_{L,\text{To}}/\bar{R_{L,\text{To}}}$, $R_{D,\text{To}}/\bar{R_{D,\text{To}}}$ and % light inhibition of *R*_D,To_ measurements in Fig. 1 that are driven by factors other than the measured temperature.
**Table S3** Model comparison between generalized additive models fitted across all $R_{L,\text{To}}/\bar{R_{L,\text{To}}}$, $R_{D,\text{To}}/\bar{R_{D,\text{To}}}$ or the light inhibition of *R*_D,To_ measurements from Australia and Denmark (across climates) with time as the predictor variable (model 1) and a model where climate (across climates) or PFT (across PFTs) was added as a covariate (model 2) or an interaction term (model 3).
**Table S4** Mean and variation in the data points of the five light inhibitions of *R*_D,To_ in Fig. 1(e,f).
Please note: Wiley Blackwell are not responsible for the content or functionality of any Supporting Information supplied by the authors. Any queries (other than missing material) should be directed to the *New Phytologist* Central Office.

## Data Availability

The data used in this article can be found in the supplementary Excel spreadsheet (Dataset S1) and are publicly available at: 10.6084/m9.figshare.20089256.
