# Supplementary material for: Consistent diurnal pattern of leaf respiration in the light among contrasting species and climates
Source: New Phytol. 2022 Jul 12;236(1):71–85. doi: 10.1111/nph.18330 (PMC9544685; doi:10.1111/nph.18330)
Supplement: Supplementary file 2 — Fig. S1 Example of a light response curve of measured and C i‐corrected leaf net CO2 exchange measurements plotted against photosynthetically active radiation. Fig. S2 Mean ambient temperature (°C) during the time of measuring the light response curves in Australia with fitted quadratic linear regression models depicting the maximum, mean and minimum temperature profiles. Fig. S3 Diurnal variation of leaf respiration in the light (R L,To) and leaf dark respiration (R D,To) from Australia and Denmark. Fig. S4 Leaf respiration in the light (RL,To/RL,To¯) and leaf dark respiration (RD,To/RD,To¯) measurements across Australia and Denmark plotted against the preset leaf measuring temperature during the time of measuring the light response curves of each leaf. Fig. S5 Diurnal variation of leaf respiration in the light, RL,To/RL,To¯, and leaf dark respiration, RD,To/RD,To¯, of Solanum nigrum, Eucalyptus saligna, Eucalyptus tereticornis and Eucalyptus parramattensis from Australia with fitted generalized additive models. Fig. S6 Diurnal variation of leaf respiration in the light, RL,To/RL,To¯, and leaf dark respiration, RD,To/RD,To¯, of Carya illinoinensis, Dichondra repens, Eucalyptus camaldulensis and Araujia sericifera from Australia with fitted generalized additive models. Fig. S7 Diurnal variation of leaf respiration in the light, RL,To/RL,To¯, and leaf dark respiration, RD,To/RD,To¯, of Malus domestica, Liriodendron tulipifera and Platanus acerifolia from Australia with fitted generalized additive models. Fig. S8 Diurnal variation of leaf respiration in the light, RL,To/RL,To¯, and leaf dark respiration, RD,To/RD,To¯,of Betula pendula, Quercus robur, Fraxinus excelsior and Salix cinerea from Denmark with fitted generalized additive models. Fig. S9 Diurnal variation of leaf respiration in the light, RL,To/RL,To¯, and leaf dark respiration, RD,To/RD,To¯, of Alnus viridis, Alnus glutinosa, Helianthus annus and Corylus avellana from Denmark with fitted generalized additiv [file NPH-236-71-s001.pdf]

Supporting information
Article title: Consistent diurnal pattern of leaf respiration in the light among contrasting species and climates
Authors: Andreas H Faber, Kevin L Griffin, Mark G Tjoelker, Majken Pagter, Jinyan Yang, Dan Bruhn
Article acceptance date: 12 June 2022

The following is included as supporting information to this article:

Table S1: Measured species in Denmark and Australia with individuals from three different plant functional types (PFTs) (deciduous, herbaceous and evergreen).

Table S2: The total diurnal variation (%) of GAMs fitted to $R_{L,To}/\overline{R_{L,To}}$, $R_{D,To}/\overline{R_{D,To}}$ and the % light inhibition of $R_{D,To}$ measurements in Fig. that are driven by other factors than the measuring temperature.

Table S3: The model comparison between GAMs fitted across all $R_{L,To}/\overline{R_{L,To}}$, $R_{D,To}/\overline{R_{D,To}}$ or the light inhibition of $R_{D,To}$ measurements from Australia and Denmark (across climates) with time as the predictor variable (model 1) and a model where climate (across climates) or PFT (across PFTs) was added as a covariate (model 2) or an interaction term (model 3).

Table S4: The mean and variation in the data points of the 5 light inhibition of $R_{D,To}$ in Fig. **1e,f**.

Fig. S1: Example of a light response curve of measured and C_i corrected leaf net CO_2 exchange measurements plotted against the photosynthetically active radiation.

Fig. S2: The mean ambient temperature (°C) during the time of measuring the light response curves in Australia with fitted quadratic linear regression models depicting the maximum, mean and minimum temperature profiles.

Fig. S3: The diurnal variation of leaf respiration in the light $R_{L,To}$ and leaf dark respiration $R_{D,To}$ from Australia and Denmark.

Fig. S4: Leaf respiration in the light $R_{L,To}/\overline{R_{L,To}}$ and leaf dark respiration $R_{D,To}/\overline{R_{D,To}}$ measurements across Australia and Denmark plotted against the pre-set leaf measuring temperature during the time of measuring the light response curves of each leaf.

Fig. S5: The diurnal variation of leaf respiration in the light, $R_{L,To}/\overline{R_{L,To}}$ and leaf dark respiration, $R_{D,To}/\overline{R_{D,To}}$ of *Solanum nigrum*, *Eucalyptus saligna*, *Eucalyptus tereticornis* and *Eucalyptus parramattensis* Australia with fitted GAMs.

Fig. S6: The diurnal variation of leaf respiration in the light, $R_{L,To}/\overline{R_{L,To}}$ and leaf dark respiration, $R_{D,To}/\overline{R_{D,To}}$ of *Carya illinoinensis*, *Dichondra repens*, *Eucalyptus camaldulensis* and *Araujia sericifera* from Australia with fitted GAMs.

Fig. S7: The diurnal variation of leaf respiration in the light, $R_{L,To}/\overline{R_{L,To}}$ and leaf dark respiration, $R_{D,To}/\overline{R_{D,To}}$ of *Malus domestica*, *Liriodendron tulipifera* and *Platanus acerifolia* from Australia with fitted GAMs.

Fig. S8: The diurnal variation of leaf respiration in the light, $R_{L,To}/\overline{R_{L,To}}$ and leaf dark respiration, $R_{D,To}/\overline{R_{D,To}}$ of *Betula pendula, Quercus robur*, *Fraxinus excelsior* and *Salix cinerea* from Denmark with fitted GAMs.

Fig. S9: The diurnal variation of leaf respiration in the light, $R_{L,To}/\overline{R_{L,To}}$ and leaf dark respiration, $R_{D,To}/\overline{R_{D,To}}$ of *Alnus viridis*, *Alnus glutinosa*, *Helianthus annus* and *Corylus avellana* from Denmark with fitted GAMs.

Fig. S10: The diurnal variation of leaf respiration in the light, $R_{L,To}/\overline{R_{L,To}}$ and leaf dark respiration, $R_{D,To}/\overline{R_{D,To}}$ of *Cornus sanguinea* and *Malus sylvestris* from Denmark with fitted GAMs.

Fig. S11: A_{gross} at the 100 µmol photons m^{-2} s^{-1} irradiance level conducted in Australia and Denmark plotted against g_{sw} at the 100 µmol photons m^{-2} s^{-1} irradiance level.

Fig. S12: a) the light inhibition of respiration measurements conducted in Australia and Denmark plotted against A_{gross} at the 100 µmol photons m^{-2} s^{-1} irradiance level. b) the light inhibition of respiration measurements conducted in Australia Denmark plotted against the ambient light intensity.

Fig. S13: Leaf respiration in the light ($R_{L,To}/\overline{R_{L,To}}$) and leaf dark respiration ($R_{D,To}/\overline{R_{D,To}}$) measurements conducted in Australia plotted against the recorded mean ambient temperature (°C) during the time of measuring the light response curves.

Fig. S14: Leaf respiration in the light ($R_{L,To}/\overline{R_{L,To}}$) and leaf dark respiration ($R_{D,To}/\overline{R_{D,To}}$) measurements conducted in Australia plotted against the recorded mean ambient VPD (kPa) during the time of measuring the light response curves.

Fig. S15: Temporal quadratic linear regression models of respiration in the light at constant temperature ($R_{L,To}$) and maximum, mean and minimum temperature variation models of respiration in the light at varying temperature ($R_{L,T}$) for Australia and Denmark, respectively.

Dataset S1: Excel sheet (see separate file "Dataset S1") containing the data used in this study including a guide on how to calculate $R_{L,T}$ throughout the day by taking into account the temporal variation in $R_{L,To}$ using the supplemented R script (see separate file "Notes S2").

Notes S1 - supplementary site description: Precipitation and temperature data from 2011-2021 and during the time of data collection for the region covering the Danish study sites and the study site in Australia.

Notes S2: R code (see separate file "Notes S2") to calculate temporal patterns of $R_{L,T}$ while accounting for temporal variations in $R_{L,To}$. The R code can be run using the R version 4.1.0 (**R Core Team. 2021**. R: A language and environment for statistical computing. R foundation for statistical computing. v.4.1.0. Vienna, Austria.) with RStudio version 1.4.1717 (**R Studio Team. 2021.** *RStudio: integrated development environment for R*. v.1.4.1717. Boston, MA, USA: RStudio, PBC.). A guide for calculating these predictions can be found in the supplemented excel sheet (see separate file "Dataset S1").

Table S1 Measured species in Denmark and Australia with individuals from three different plant functional types (PFTs) (deciduous, herbaceous and evergreen). The height of the measured plants is denoted in meters (m).

Year	Species	PFT	Height (m)
**Denmark**			
2019	*Alnus glutinosa* (L.) Gaertn.	Deciduous	0.5
	Alnus viridis (Chaix) DC.	Deciduous	2
	Betula pendula (Roth)	Deciduous	1.5
	Cornus sanguinea (L.)	Deciduous	1
	Corylus avellana (L.)	Deciduous	5
	Helianthus annuus (L.)	Herbaceous	1.5
	Malus sylvestris (L.) Mill.	Deciduous	2 - 3
	Quercus robur (L.)	Deciduous	6
2021	*Salix cinerea* (L.)	Deciduous	2
	Fraxinus excelsior (L.)	Deciduous	1.5
**Australia**			
2020	*Araujia sericifera* (Brot.)*	Herbaceous	0.1
	Carya illinoinensis (Wangenh.) K. Koch.	Deciduous	0.3 - 0.5
	Dichondra repens (J.R. Frost. & G. Frost.)*	Herbaceous	0.1
	Eucalyptus camaldulensis (Dehnh.)	Evergreen	4
	Eucalyptus parramattensis (E.C. Hall.)	Evergreen	1 – 4
	Eucalyptus saligna (Sm.)	Evergreen	4
	Eucalyptus tereticornis (Sm.)	Evergreen	2 - 4
	Liriodendron tulipifera (L.)*	Deciduous	1.5
	Malus domestica (Borkh.)*	Deciduous	1.5
	Platanus acerifolia (Aiton.) Willd.	Deciduous	6
	Solanum nigrum (L.)*	Herbaceous	0.3 - 0.4

* Watered with clean wastewater every fourth day during the study period.

Table S2 The total diurnal variation (%) of GAMs fitted to $R_{L,To}/\overline{R_{L,To}}$, $R_{D,To}/\overline{R_{D,To}}$ and the % light inhibition of $R_{D,To}$ measurements in Fig. **1** that are driven by other factors than the measuring temperature. The variation was calculated from the difference between the minimum and maximum predicted values of $R_{L,To}/\overline{R_{L,To}}$, $R_{D,To}/\overline{R_{D,To}}$ and the % light inhibition of $R_{D,To}$, divided by the maximum GAM predicted values.

Measurement	Across climates	Australia	Denmark	Deciduous	Evergreen	Herbaceous
$R_{L,To}/\overline{R_{L,To}}$	38	45	33	40	42	49
$R_{D,To}/\overline{R_{D,To}}$	12	24	2	21	23	28
Light inhibition	47	63	32	58	70	61

Table S3 The model comparison between GAMs fitted across all $R_{L,To}/\overline{R_{L,To}}$, $R_{D,To}/\overline{R_{D,To}}$ or the light inhibition of $R_{D,To}$ measurements from Australia and Denmark (across climates) with time as the predictor variable (model 1) and a model where climate (across climates) or PFT (across PFTs) was added as a covariate (model 2) or an interaction term (model 3). The model with the lowest Akaike's information criterion (AIC) value is denoted with asterisk. The PFTs analysis was performed only on the data from Australia. The degrees of freedom (df) are denoted for each fitted model.

	**Across climates –** $R_{L,To}/\overline{R_{L,To}}$		**Across climates –** $R_{D,To}/\overline{R_{D,To}}$		**Across climates – light inhibition of** $\boldsymbol{R_{D,To}}$	
**Mode l**	**df**	**AIC**	**df**	**AIC**	**df**	**AIC**
1	5	-503.6970	5	-1166.465	5	4294.632
2	6	-495.5306	6	-1157.723	6	4219.554*
3	8	-519.0516*	8	-1261.866*	8	4222.017
	**Across PFTs –** $R_{L,To}/\overline{R_{L,To}}$		**Across PFTs –** $R_{D,To}/\overline{R_{D,To}}$		**Across PFTs – light inhibition of** $\boldsymbol{R_{D,To}}$	
1	5	-336.2825*	5	-605.5268*	5	1990.156
2	7	-321.2026	7	-588.7830	7	1981.970*
3	10	-320.3561	10	-584.4894	10	1987.970

Table S4 The mean and variation in the data points of the % light inhibition of $R_{D,To}$ in Fig. **1e** (Australia and Denmark and across both climates), and **1f** (the deciduous, evergreen and herbaceous species measured in Australia).

**Estimate**	**Across climates**	**Australia**	**Denmark**	**Deciduous**	**Evergreen**	**Herbaceous**
Mean	32%	18%	45%	20%	15%	21%
Variation	-9 – 95%	-9 – 60%	10 – 95%	3 – 53%	-1 – 60%	-9 – 60%

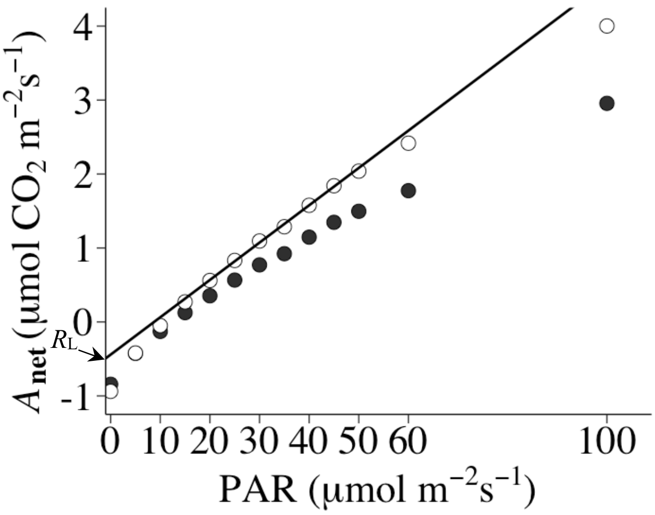

Fig. S1. Example of a light response curve of measured (closed circles) and C_i corrected (open circles) leaf net CO_2 exchange measurements (A_{net}, µmol CO_2 m^{-2} s^{-1}) plotted against the photosynthetically active radiation (PAR, µmol photons m^{-2} s^{-1}). The C_i corrected A_{net} values are plotted as V_J - R_L, where V_J and R_L were calculated with the iterative approach according to **Kirschbaum MUF, Farquhar GD**. **1987**. Investigation of the CO_2 Dependence of Quantum Yield and Respiration in *Eucalyptus pauciflora*. *Plant physiology* **83**: 1032–1036. https://doi.org/10.1104/pp.83.4.1032. The break from linearity at 20 µmol photons m^{-2} s^{-1} illustrates the Kok effect. R_L was estimated by the intercept of the linear regression fitted to the linear region above the Kok effect of the C_i corrected data. R_D is the dark respiration estimated directly from the CO_2 efflux following 10 minutes at 0 µmol photons m^{-2} s^{-1}. The data of this example was obtained from a single leaf measurement on Red gum (*Eucalyptus parramattensis*) from the Hawkesbury Forest Experiment site, Richmond, NSW, Australia.

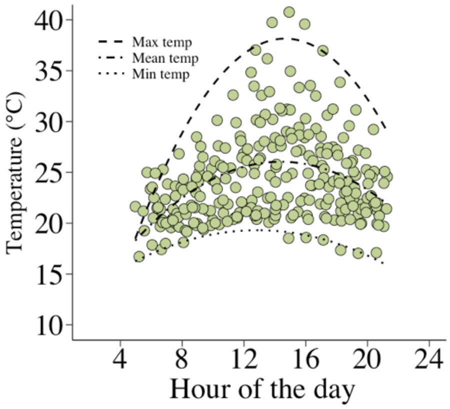

Fig. S2. The mean ambient temperature (°C) during the time of measuring the light response curves in Australia (n = 270). The maximum, mean and minimum temperature quadratic linear regression models ($y_i = -6.833991 + 6.136696time_i - 0.209268time_i{}^2 + \varepsilon_i, y_i = 7.939280 + 2.517968time_i - 0.087554time_i{}^2 + \varepsilon_i$ and $y_i = 11.184457 + 1.262541time_i - 0.049048time_i{}^2 + \varepsilon_i$, respectively) are fitted through the maximum, mean and minimum temperature recorded within two-hour intervals from 4 AM to 22 PM. The data were provided by the HFE climate station in Australia.

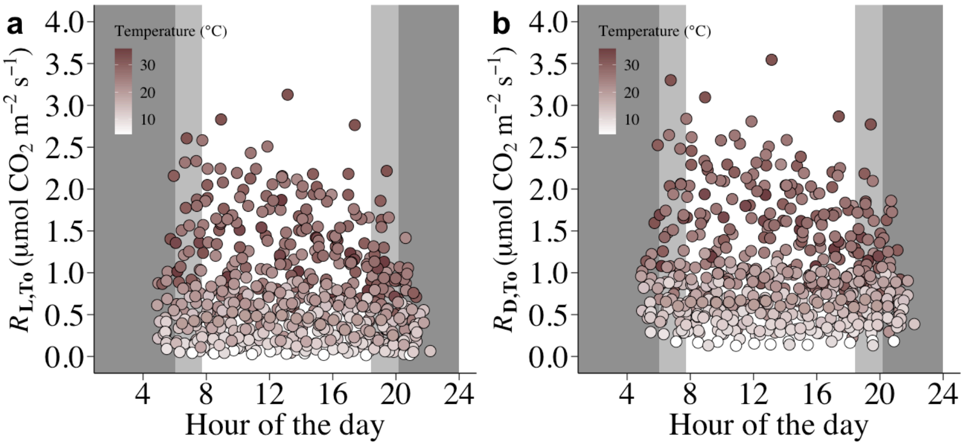

Fig. S3. The diurnal variation of leaf respiration in the light a) $R_{L,To}$ (µmol CO_2 m^{-2} s^{-1}) and leaf dark respiration b) $R_{D,To}$ (µmol CO_2 m^{-2} s^{-1}) from Australia and Denmark (n = 562). The colour scale denotes the extend by which the pre-set measuring temperature varied between different days (4.5-35 °C). Dark shaded areas illustrate night-time shared for all days of measuring and light shaded areas illustrate the variation in time of sunrise and sunset, respectively, between the days of measuring. The studied species can be found in table S1.

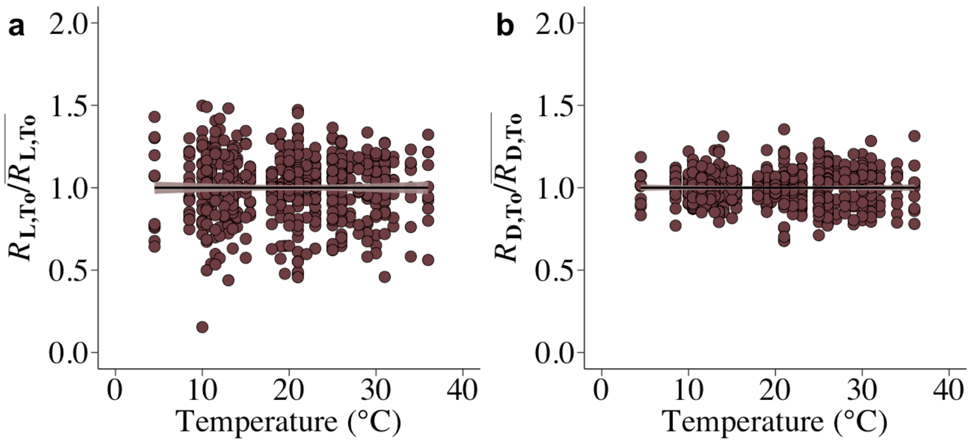

Fig. S4. Leaf respiration in the light a) $R_{L,To}/\overline{R_{L,To}}$ and leaf dark respiration b) $R_{D,To}/\overline{R_{D,To}}$ measurements across Australia and Denmark (n = 562) plotted against the pre-set leaf measuring temperature (°C) during the time of measuring the light response curves of each leaf. Linear regression models with 95% point-wise confidence intervals are fitted to the a) $R_{L,To}/\overline{R_{L,To}}$ ($y_i = 1 + 2.143 * 10^{-18} temperature_i + \varepsilon_i$, r^2 = 1.234667*10^{-29}, $P > 0.05$), b) $R_{D,To}/\overline{R_{D,To}}$ ($y_i = 1 + 1.607 * 10^{-18} temperature_i + \varepsilon_i$, r^2 = 3.505181*10^{-30}, $P > 0.05$). The studied species can be found in table S1.

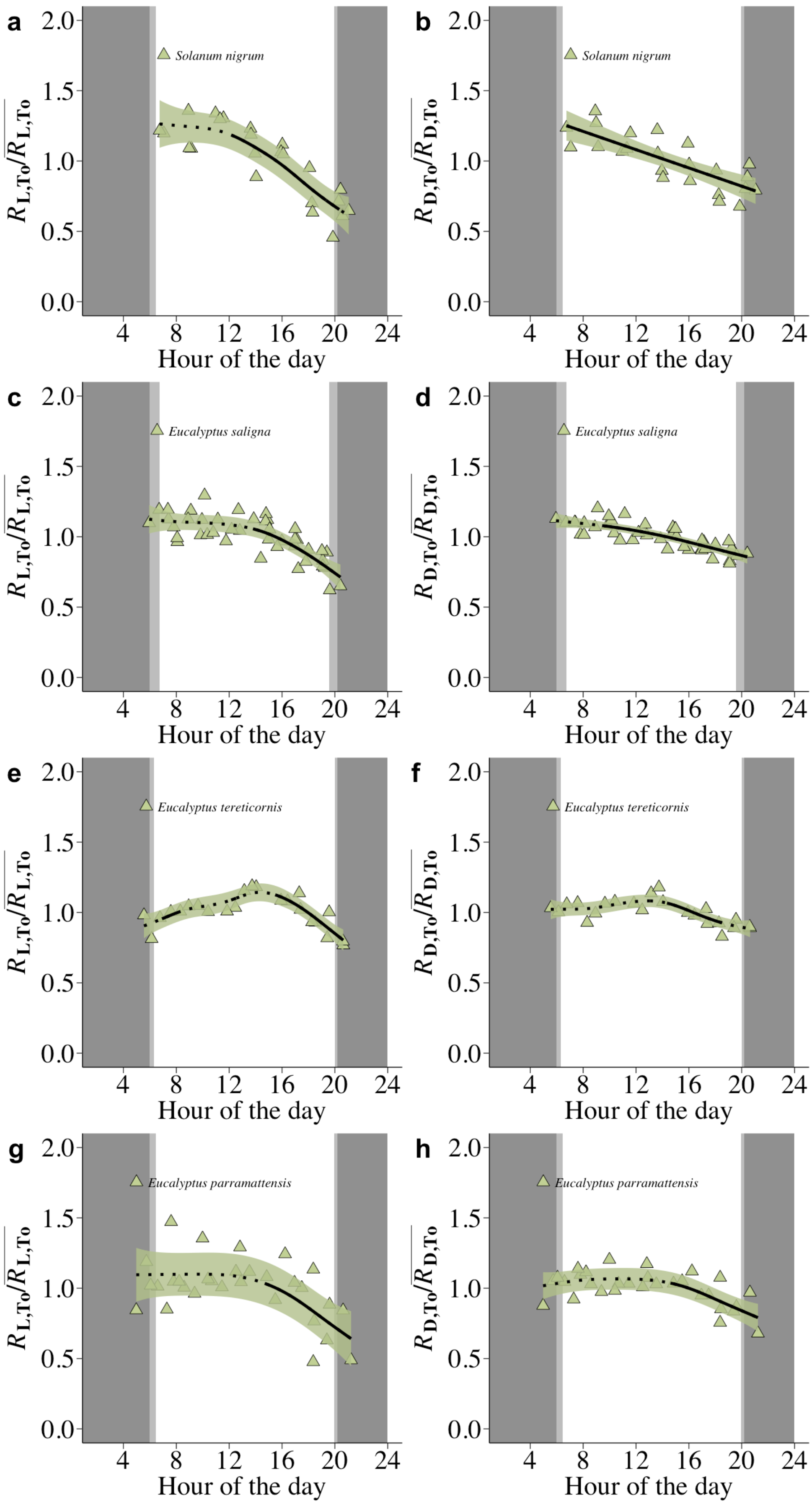
a
b
c
d
e
f
g
h
Solanum nigrum
Eucalyptus saligna
Eucalyptus tereticornis
Eucalyptus parramattensis
$R_{L,To}/\overline{R_{L,To}}$
$R_{D,To}/\overline{R_{D,To}}$
Hour of the day
0.0
0.5
1.0
1.5
2.0
4
8
12
16
20
24

Fig. S5. The diurnal variation of a), c), e) and g) leaf respiration in the light, $R_{L,To}/\overline{R_{L,To}}$ and b), d), f) and h) leaf dark respiration, $R_{D,To}/\overline{R_{D,To}}$. GAMs with 95% point-wise confidence intervals are fitted across measurements from plant species in Australia in a and b) *Solanum nigrum* (n = 23), c and d) *Eucalyptus saligna* (n = 42), e and f) *Eucalyptus tereticornis* (n = 23) and g and h) *Eucalyptus parramattensis* (n = 30). Significant variations in the diurnal variation of $R_{L,To}/\overline{R_{L,To}}$ and $R_{D,To}/\overline{R_{D,To}}$ are indicated by the solid portions of the fitted GAMs while dotted portions illustrate non-significant variations. Dark shaded areas illustrate night-time shared for all days of measuring and light shaded areas illustrate the variation in time of sunrise and sunset between the days of measuring.

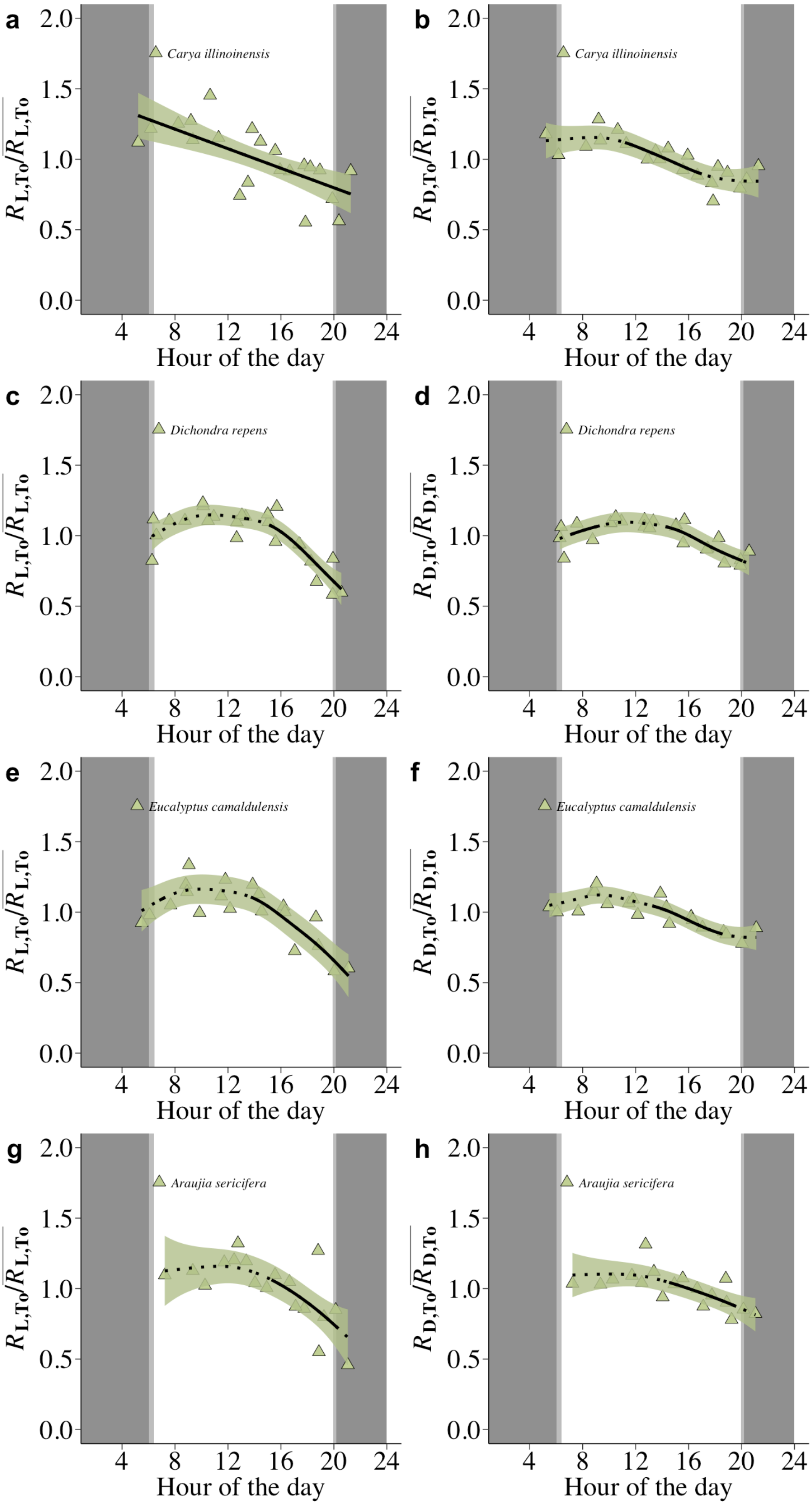
a
b
c
d
e
f
g
h
Carya illinoinensis
Dichondra repens
Eucalyptus camaldulensis
Araujia sericifera
$R_{L,To}/\overline{R_{L,To}}$
$R_{D,To}/\overline{R_{D,To}}$
Hour of the day
0.0
0.5
1.0
1.5
2.0
4
8
12
16
20
24

Fig. S6. The diurnal variation of a), c), e) and g) leaf respiration in the light, $R_{L,To}/\overline{R_{L,To}}$ and b), d), f) and h) leaf dark respiration, $R_{D,To}/\overline{R_{D,To}}$. GAMs with 95% point-wise confidence intervals are fitted across measurements from plant species in Australia in a and b) *Carya illinoinensis* (n = 21), c and d) *Dichondra repens* (n = 24), e and f) *Eucalyptus camaldulensis* (n = 20) and g and h) *Araujia sericifera* (n = 18). Significant variations in the diurnal variation of $R_{L,To}/\overline{R_{L,To}}$ and $R_{D,To}/\overline{R_{D,To}}$ are indicated by the solid portions of the fitted GAMs while dotted portions illustrate non-significant variations. Dark shaded areas illustrate night-time shared for all days of measuring and light shaded areas illustrate the variation in time of sunrise and sunset between the days of measuring.

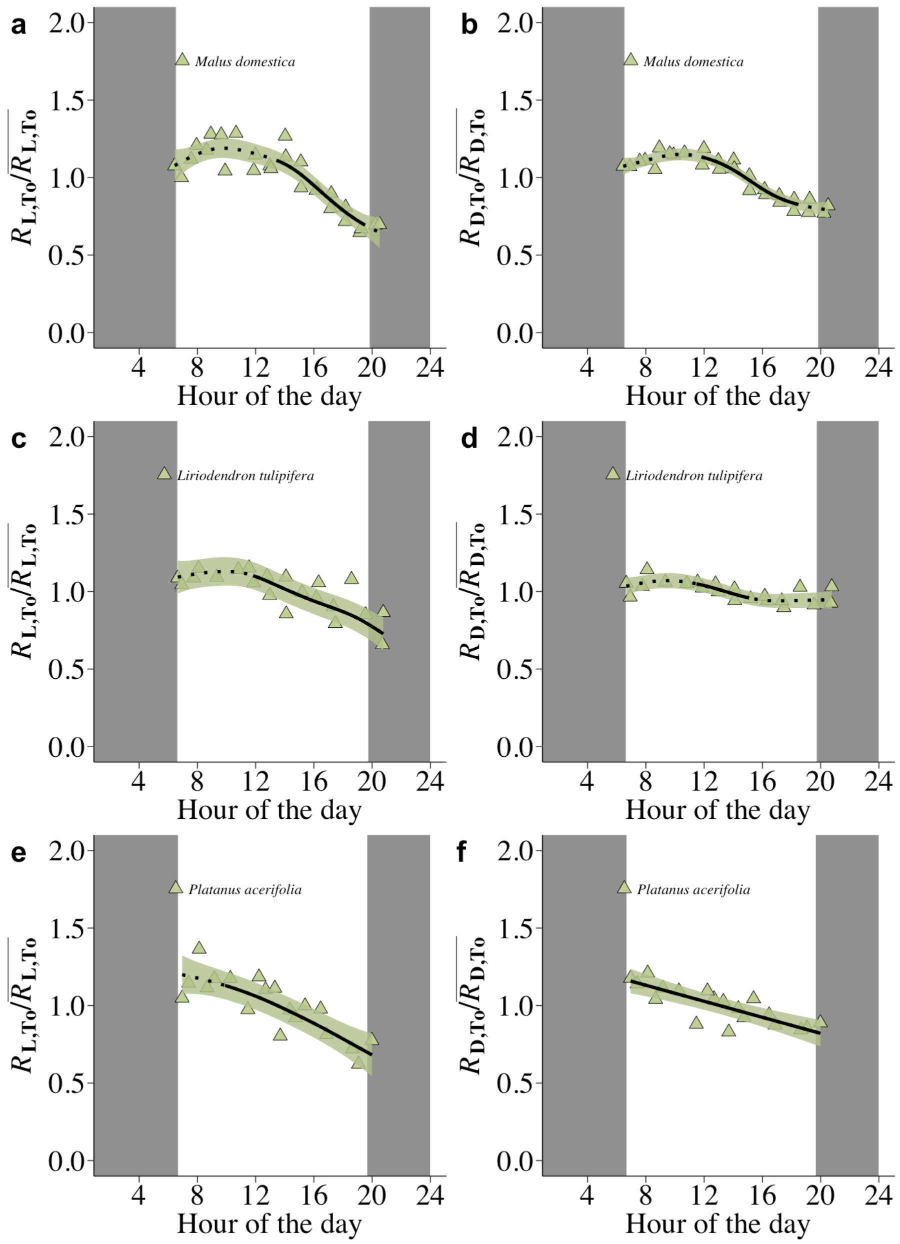

Fig. S7. The diurnal variation of a), c) and e) leaf respiration in the light, $R_{L,To}/\overline{R_{L,To}}$ and b), d) and f) leaf dark respiration, $R_{D,To}/\overline{R_{D,To}}$. GAMs with 95% point-wise confidence intervals are fitted across measurements from plant species in Australia in a and b) *Malus domestica* (n = 27), c and d) *Liriodendron tulipifera* (n = 21) and e and f) *Platanus acerifolia* (n = 19). Significant variations in the diurnal variation of $R_{L,To}/\overline{R_{L,To}}$ and $R_{D,To}/\overline{R_{D,To}}$ are indicated by the solid portions of the fitted GAMs while dotted portions illustrate non-significant variations. Dark shaded areas illustrate night-time shared for all days of measuring and light shaded areas illustrate the variation in time of sunrise and sunset between the days of measuring.

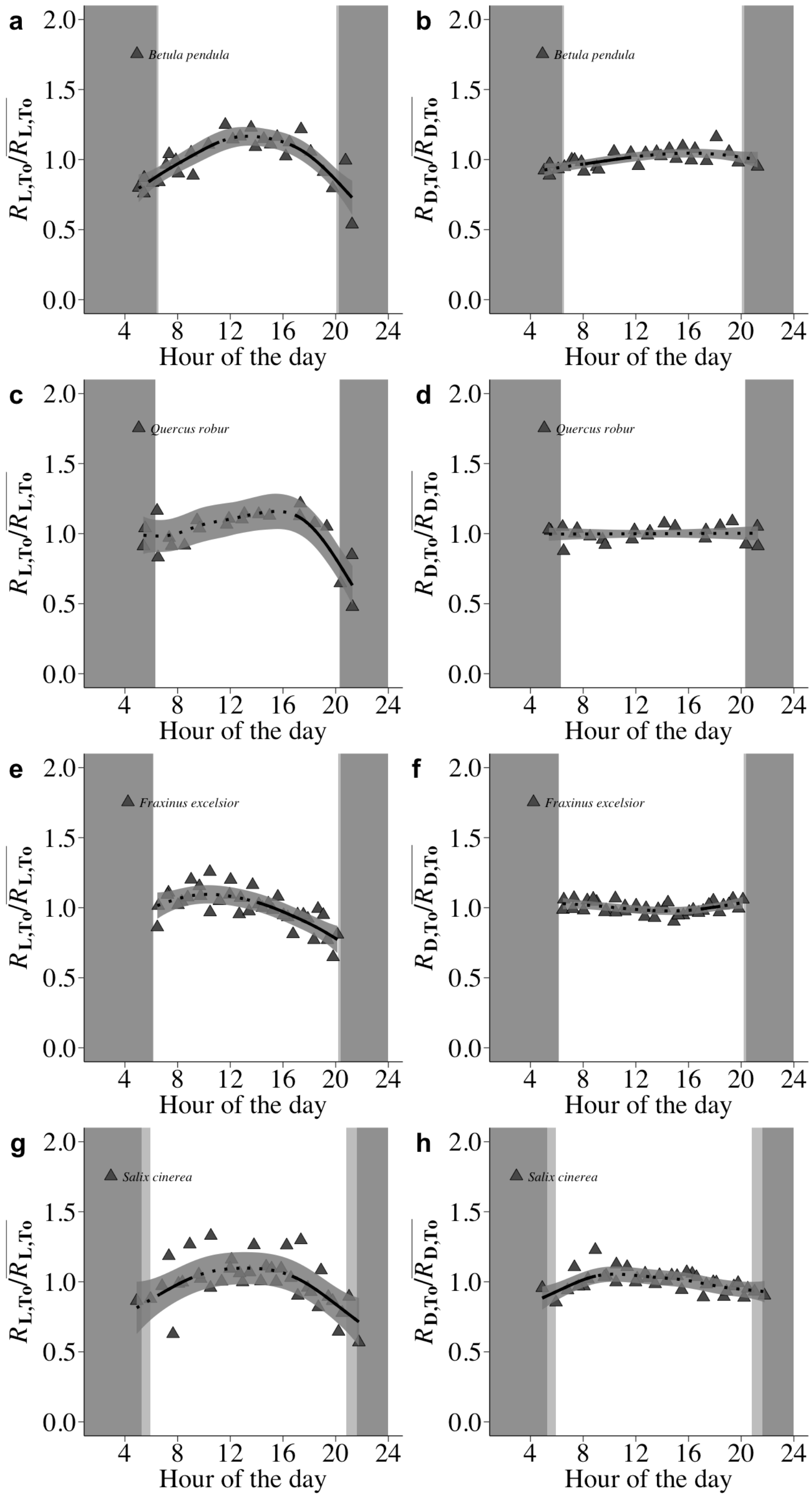
a
b
c
d
e
f
g
h
Betula pendula
Quercus robur
Fraxinus excelsior
Salix cinerea
Hour of the day

Fig. S8. The diurnal variation of a), c), e) and g) leaf respiration in the light, $R_{L,To}/\overline{R_{L,To}}$ and b), d), f) and h) leaf dark respiration, $R_{D,To}/\overline{R_{D,To}}$. GAMs with 95% point-wise confidence intervals are fitted across measurements from plant species in Denmark in a and b) *Betula pendula* (n = 28), c and d) *Quercus robur* (n = 22), e and f) *Fraxinus excelsior* (n = 37) and g and h) *Salix cinerea* (n = 38). Significant variations in the diurnal variation of $R_{L,To}/\overline{R_{L,To}}$ and $R_{D,To}/\overline{R_{D,To}}$ are indicated by the solid portions of the fitted GAMs while dotted portions illustrate non-significant variations. Dark shaded areas illustrate night-time shared for all days of measuring and light shaded areas illustrate the variation in time of sunrise and sunset between the days of measuring.

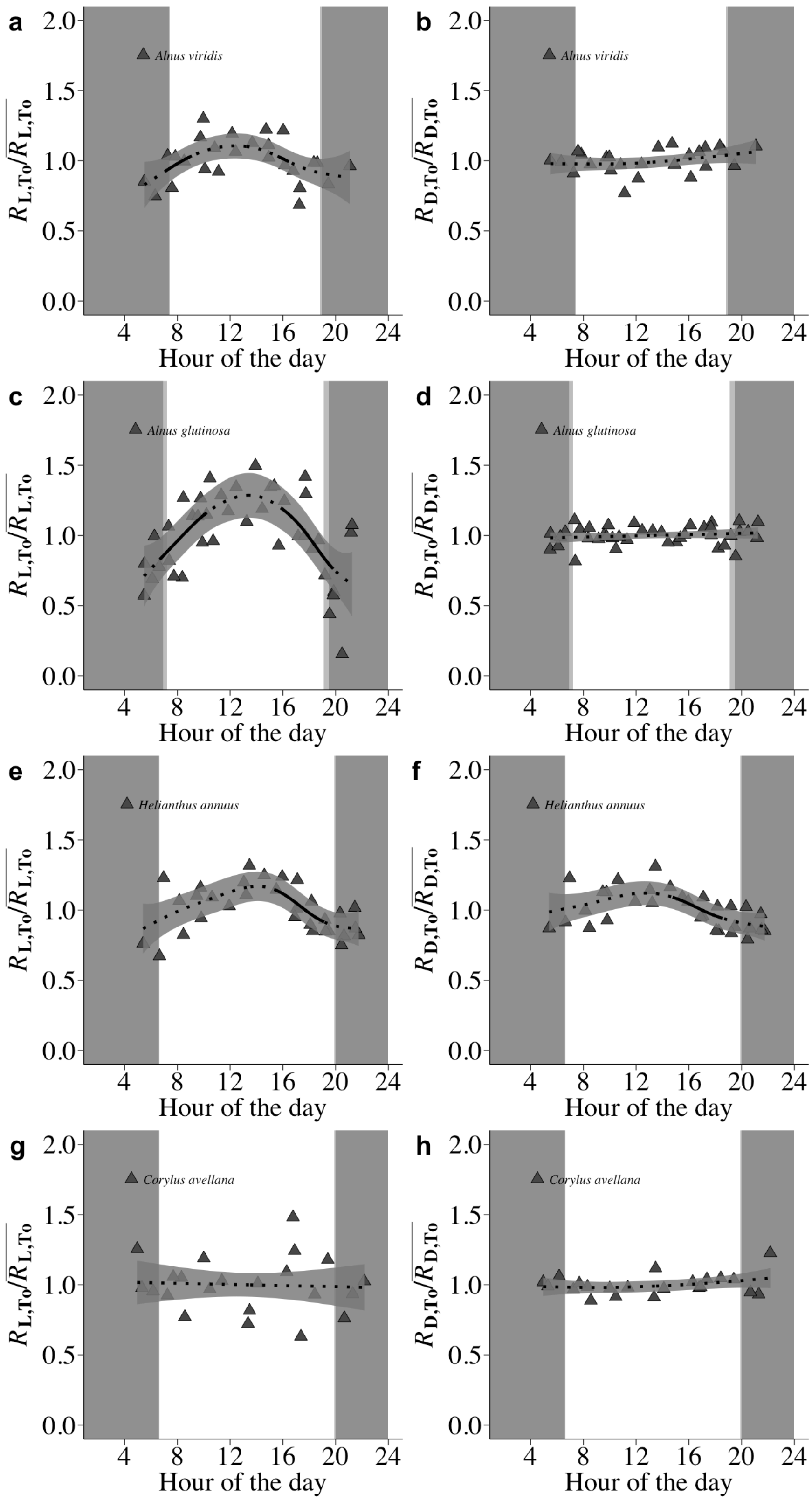

Fig. S9. The diurnal variation of a), c), e) and g) leaf respiration in the light, $R_{L,To}/\overline{R_{L,To}}$ and b), d), f) and h) leaf dark respiration, $R_{D,To}/\overline{R_{D,To}}$. GAMs with 95% point-wise confidence intervals are fitted across

measurements from plant species in Denmark in a and b) *Alnus viridis* (n = 26), c and d) *Alnus glutinosa* (n = 40), e and f) *Helianthus annus* (n = 31) and g and h) *Corylus avellana* (n = 22). Significant variations in the diurnal variation of $R_{L,To}/\overline{R_{L,To}}$ and $R_{D,To}/\overline{R_{D,To}}$ are indicated by the solid portions of the fitted GAMs while dotted portions illustrate non-significant variations. Dark shaded areas illustrate night-time shared for all days of measuring and light shaded areas illustrate the variation in time of sunrise and sunset between the days of measuring.

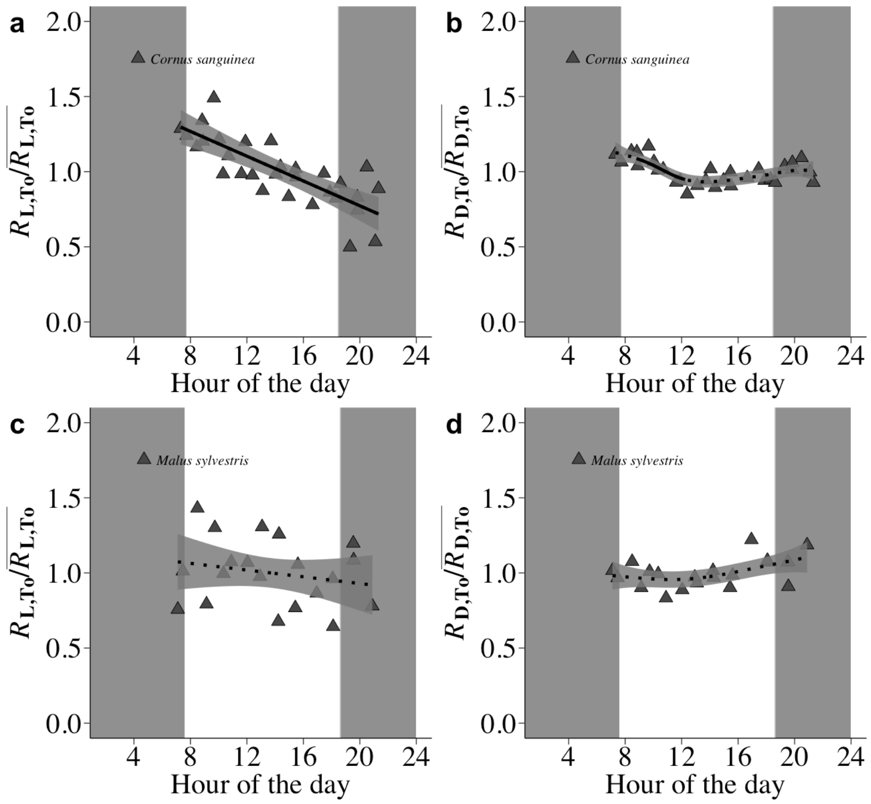

Fig. S10. The diurnal variation of a) and c) leaf respiration in the light, $R_{L,To}/\overline{R_{L,To}}$ and b) and d) leaf dark respiration, $R_{D,To}/\overline{R_{D,To}}$. GAMs with 95% point-wise confidence intervals are fitted across measurements from plant species in Denmark in a and b) *Cornus sanguinea* (n = 30) and c and d) *Malus sylvestris* (n = 20). Significant variations in the diurnal variation of $R_{L,To}/\overline{R_{L,To}}$ and $R_{D,To}/\overline{R_{D,To}}$ are indicated by the solid portions of the fitted GAMs while dotted portions illustrate non-significant variations. Dark shaded areas illustrate night-time shared for all days of measuring and light shaded areas illustrate the variation in time of sunrise and sunset between the days of measuring.

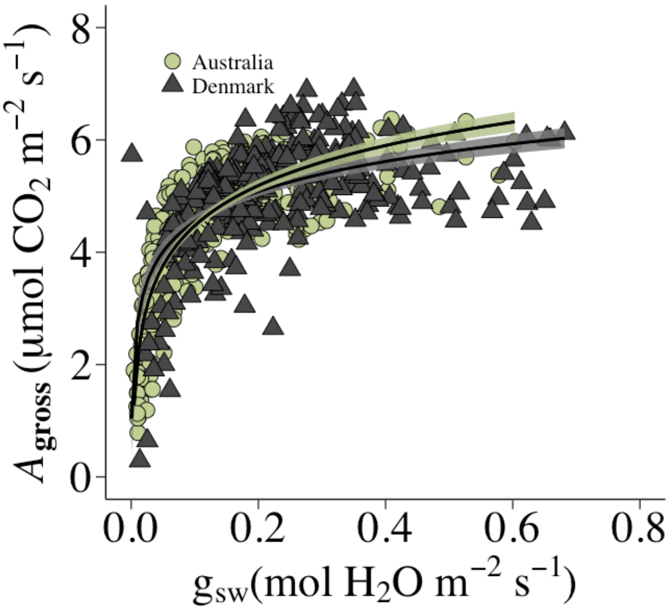

Fig. S11. A_{gross} (µmol CO_2 m^{-2} s^{-1}) at the 100 µmol photons m^{-2} s^{-1} irradiance level conducted in Australia (green circles, n = 268) and Denmark (dark triangles, n = 294) plotted against g_{sw} (mol H_2O m^{-2} s^{-1}) at the 100 µmol photons m^{-2} s^{-1} irradiance level. Logarithmic models with 95% point-wise confidence intervals are fitted to the measurements from Australia ($y_i = 6.84355 + 1.02978\ln(g_{sw_i}) + \varepsilon_i$, $r^2 = 0.71$, $P < 0.05$) and Denmark ($y_i = 6.32467 + 0.77324\ln(g_{sw_i}) + \varepsilon_i$, $r^2 = 0.35$, $P < 0.05$). The studied species can be found in table S1.

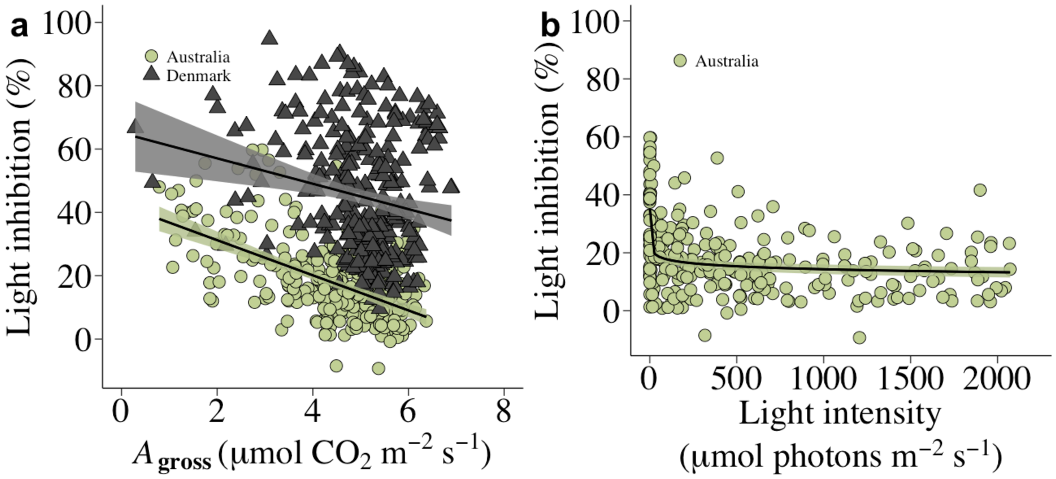

Fig. S12. The light inhibition of leaf respiration (1-($R_{L,To}$/$R_{D,To}$)100) a) measurements conducted in Australia (green circles, n = 268) and Denmark (dark triangles, n = 294) plotted against A_{gross} at the 100 µmol photons m^{-2} s^{-1} irradiance level. b) the light inhibition of leaf respiration measurements conducted in Australia plotted against the recorded mean ambient light intensity (µmol photons m^{-2} s^{-1}) during the time of measuring the light response curves. Linear regression models with 95% point-wise confidence intervals a) ($y_i = 42.3028 - 5.5454A_{gross_i} + \varepsilon_i$, $r^2 = 0.30$, $P < 0.05$ and $y_i = 65.067 - 4.008A_{gross_i} + \varepsilon_i$, $r^2 = 0.04$, $P < 0.05$) are fitted to the measurements in Australia and Denmark, respectively. A logarithmic regression model with a 95% point-wise confidence interval b) ($y_i = 24.4616 - 1.4676\ln(light_i) + \varepsilon_i$, $r^2 = 0.21$, $P < 0.05$) are fitted to the measurements in Australia. The studied species can be found in table S1.

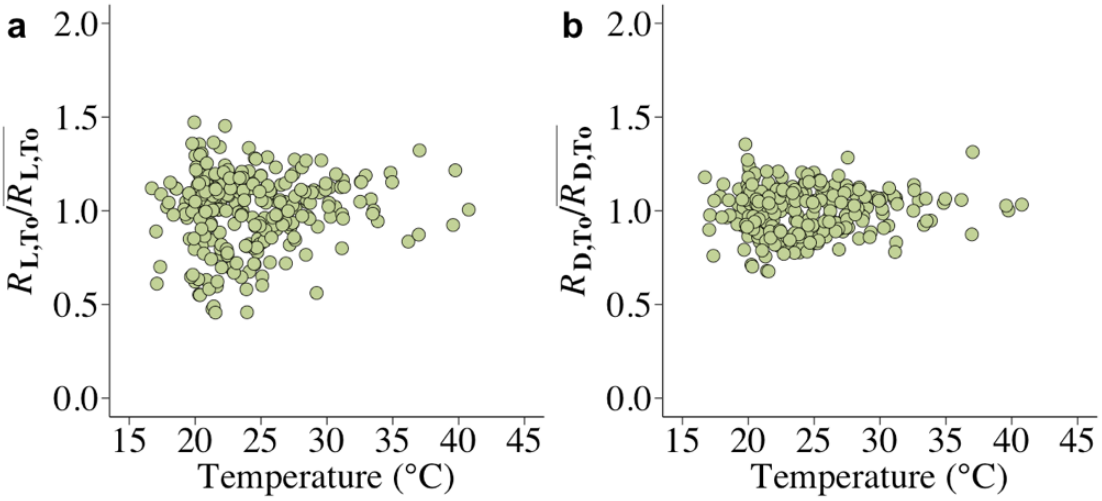

Fig. S13. Leaf respiration in the light a) ($R_{L,To}/\overline{R_{L,To}}$) and leaf dark respiration b) ($R_{D,To}/\overline{R_{D,To}}$) measurements conducted in Australia (n = 268) plotted against the recorded mean ambient temperature (°C) during the time of measuring the light response curves. The studied species can be found in table S1.

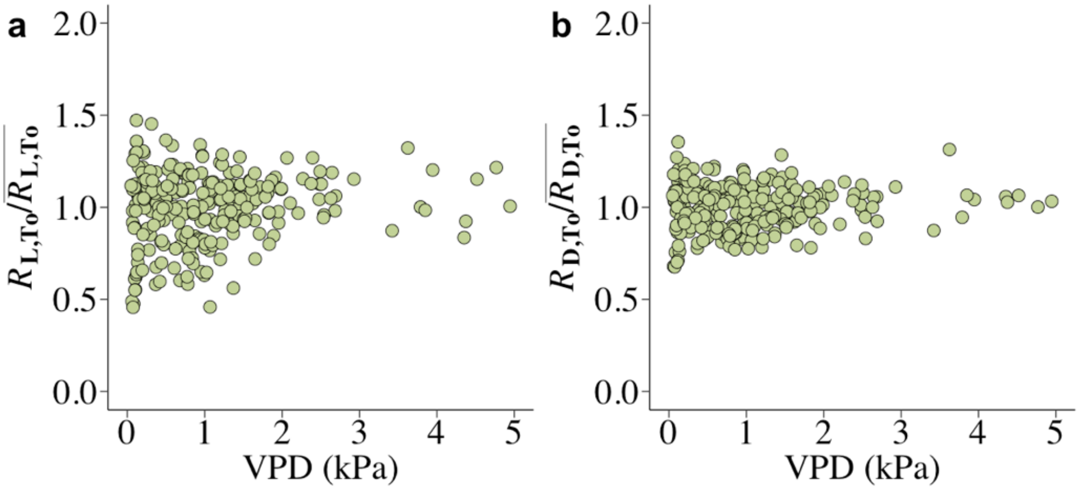

Fig. S14. Leaf respiration in the light a) ($R_{L,To}/\overline{R_{L,To}}$) and leaf dark respiration b) ($R_{D,To}/\overline{R_{D,To}}$) measurements conducted in Australia (n = 268) plotted against the recorded mean ambient VPD (kPa) during the time of measuring the light response curves. The studied species can be found in table S1.

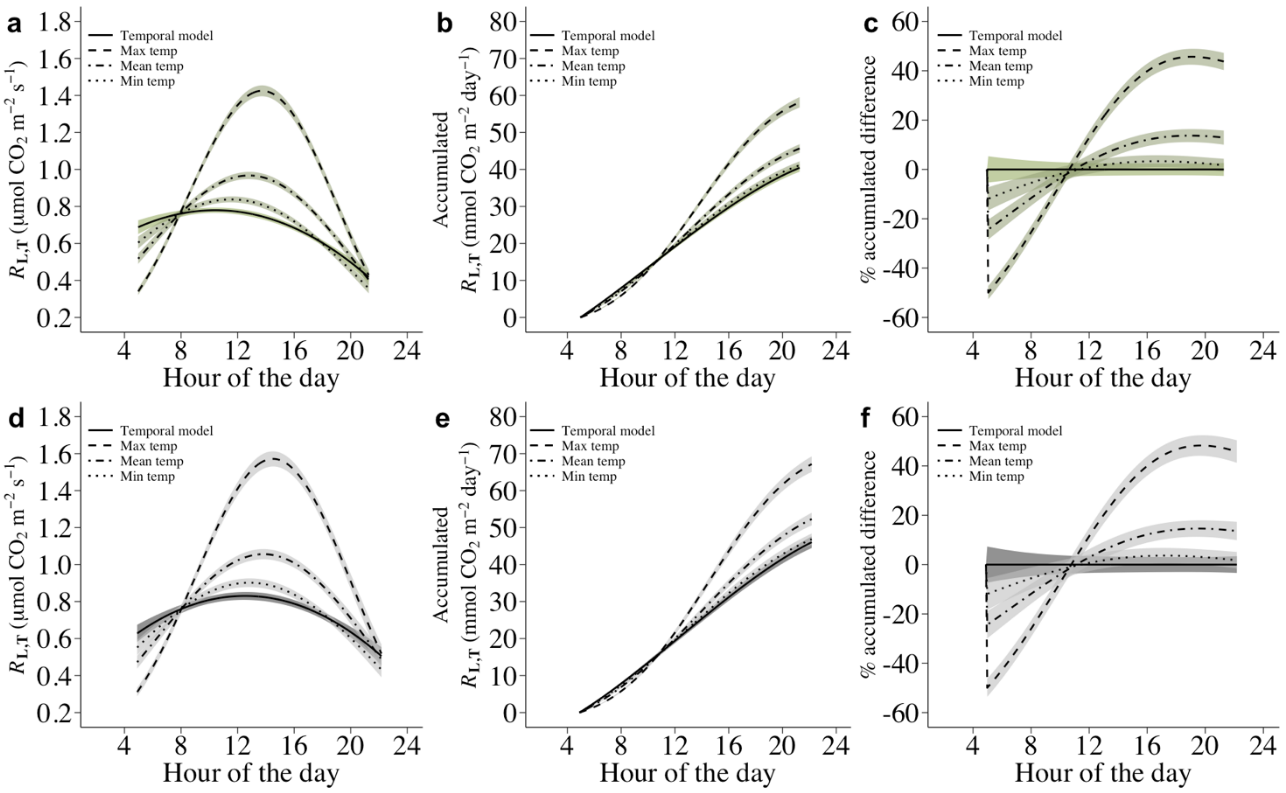

Fig. S15. Temporal quadratic linear regression models of respiration in the light at constant temperature ($R_{L,To}$) (solid line) and maximum, mean and minimum temperature variation models of respiration in the light at varying temperature ($R_{L,T}$) (dashed, dot dashed and dotted lines, respectively) with shaded 95 % pointwise confidence bands for Australia a) and Denmark d). For the temporal quadratic linear regression models, a hypothetical $R_{L,To}$ value of 0.76 μmol CO_2 m^{-2} s^{-1} (i.e. the mean estimated $R_{L,To}$ value across all species in this study) measured at 8 AM was used to predict $R_{L,To}$ values throughout the day at a constant temperature. The maximum, mean and minimum temperature variation models are based on three temperature profiles and predict $R_{L,T}$. The predictions of $R_{L,T}$ were derived by fitting quadratic linear regression models to the $R_{L,To}/\overline{R_{L,To}}$ measurements from Australia and Denmark. Subsequently, the hypothetical $R_{L,To}$ value measured at 8 AM was used to predict $R_{L,T}$ throughout the day at different temperatures from the fitted quadratic linear regression model from Australia as: $R_{L,Ti} = (0.76((0.6487044 + 0.0936338 time_i - 0.0044895 time_i^2 + \varepsilon_i)/(0.6487044 + 0.0936338 time_x - 0.0044895 time_x^2 + \varepsilon_x)))2\char`^((T_i - T_o/10)$ and from the fitted quadratic linear regression model from Denmark as: $R_{L,Ti} = (0.76((0.3795814 + 0.1168153 time_i - 0.0046665 time_i^2 + \varepsilon_i)/(0.3795814 + 0.1168153 time_x - 0.0046665 time_x^2 + \varepsilon_x)))2\char`^((T_i - T_o/10)$, where 0.76 is the $R_{L,To}$ value

measured at 8 AM ($time_x$), ε_x is the estimated residual error at 8 AM, $R_{L,Ti}$ is the predicted rates of respiration at temperatures T_i and time points $time_i$, and ε_i is the residual errors at $time_i$. T_o is the temperature at the time where the $R_{L,To}$ value of 0.76 was measured and 2 denotes the factor by which $R_{L,Ti}$ changes for every 10°C temperature change. The temperature profiles were derived from the measured ambient temperature during the time of measuring the light response curves in Australia and can be viewed in Fig. S2. b) and e) the daily accumulated CO_2 efflux predicted by the temporal, max temp, mean temp and min temp variation models for Australia and Denmark, respectively. c) and f) percent difference between accumulated CO_2 predicted by the max temp, mean temp and min temp variation models and the temporal models for Australia and Denmark, respectively.

Notes S1 - supplementary site description

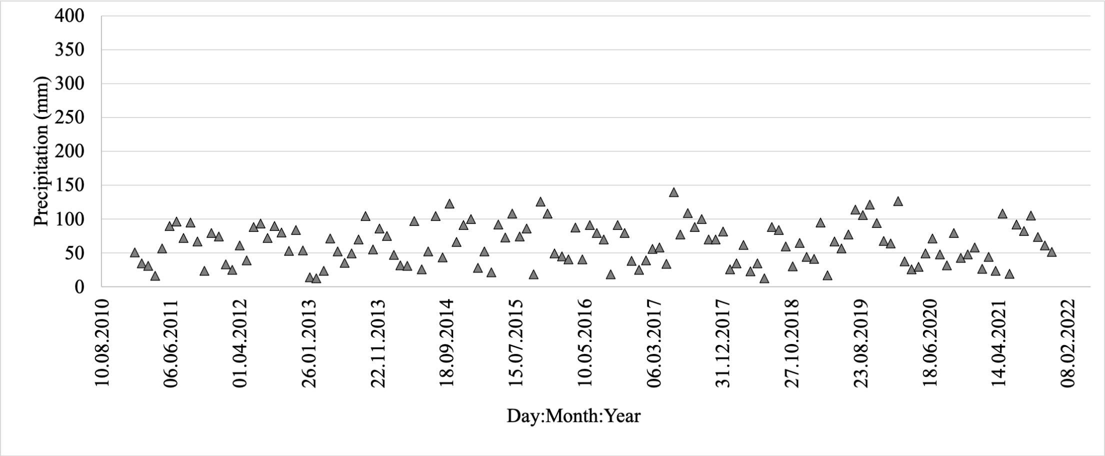

Fig. A. Total monthly precipitation (mm) from the region covering the two study sites in Denmark from 2011-2021. The data was attained from the Danish Meteorological Institute (www.DMI.dk) from the region Mariagerfjord municipality.

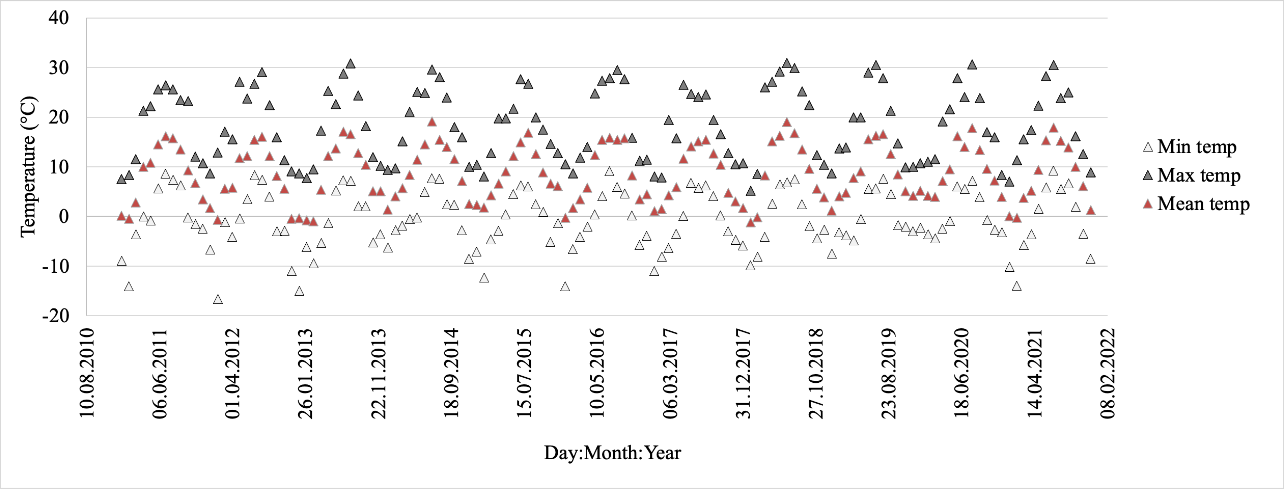

Fig. B. Monthly minimum, mean and maximum temperature (°C) from the region covering the two study sites in Denmark from 2011-2021. The data was attained from the Danish Meteorological Institute (www.DMI.dk) from the region Mariagerfjord municipality.

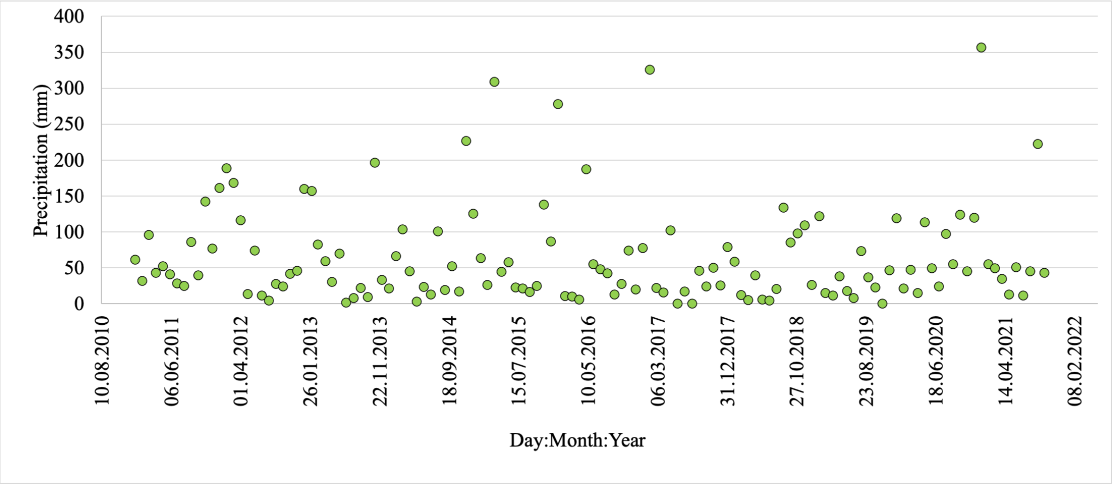

Fig. C. Total monthly precipitation (mm) from the study site in Australia from 2011-2021. The data was attained from the Australian Bureau of Meteorology (http://www.bom.gov.au).

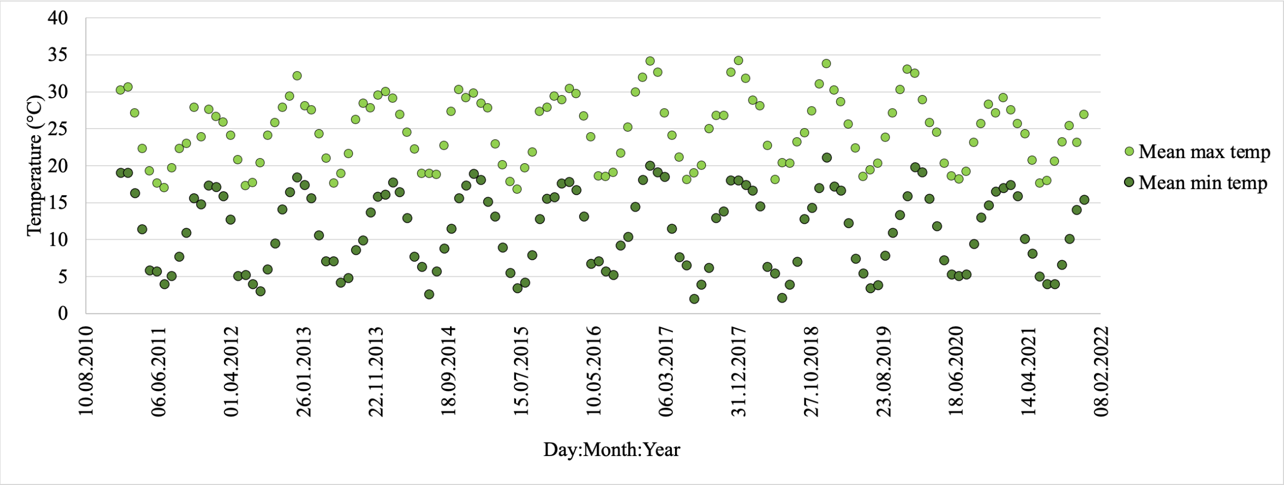

Fig. D. Monthly mean maximum and mean minimum temperature (°C) from the study site in Australia from 2011-2021. The data was attained from the Australian Bureau of Meteorology (http://www.bom.gov.au).

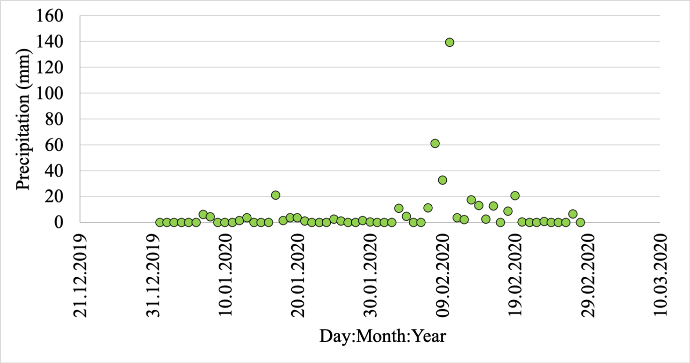

Fig. E. Total daily precipitation (mm) during the time of data collection from the study site in Australia from 01.01.2020-28.02.2020 (Day:Month:Year). The data was attained from the Australian Bureau of Meteorology (http://www.bom.gov.au).

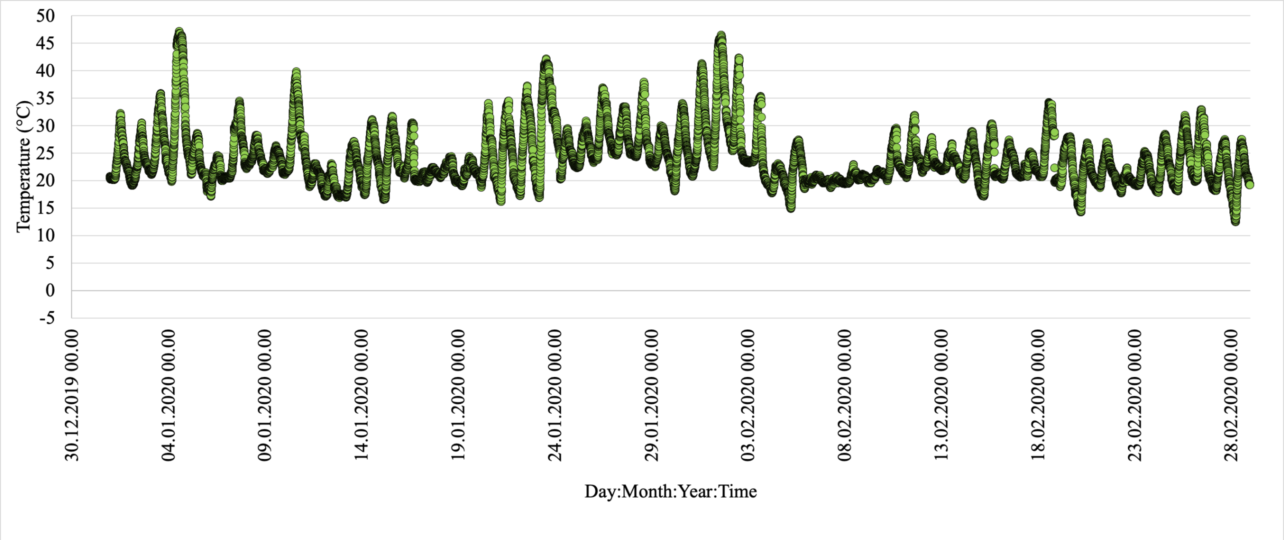

Fig. F. Mean temperature (°C) every 10 minutes during the time of data collection from the study site in Australia from 01.01.2020-28.02.2020 (Day:Month:Year). The data was attained from the Australian Bureau of Meteorology (http://www.bom.gov.au).

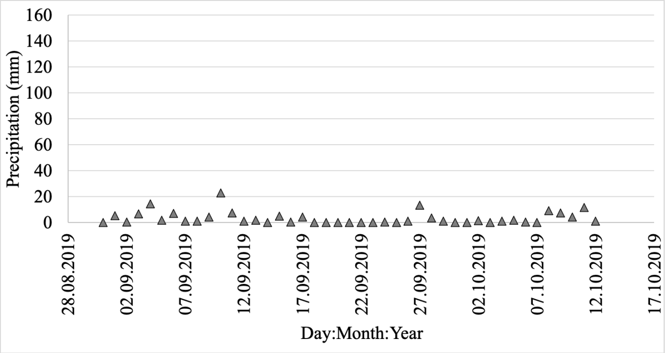

Fig. G. Total daily precipitation (mm) during the time of data collection from the region covering the two study sites in Denmark 31.08.2019-12.10.2019 (Day:Month:Year). The data was attained from the Danish Meteorological Institute (www.DMI.dk) from the region Mariagerfjord municipality.

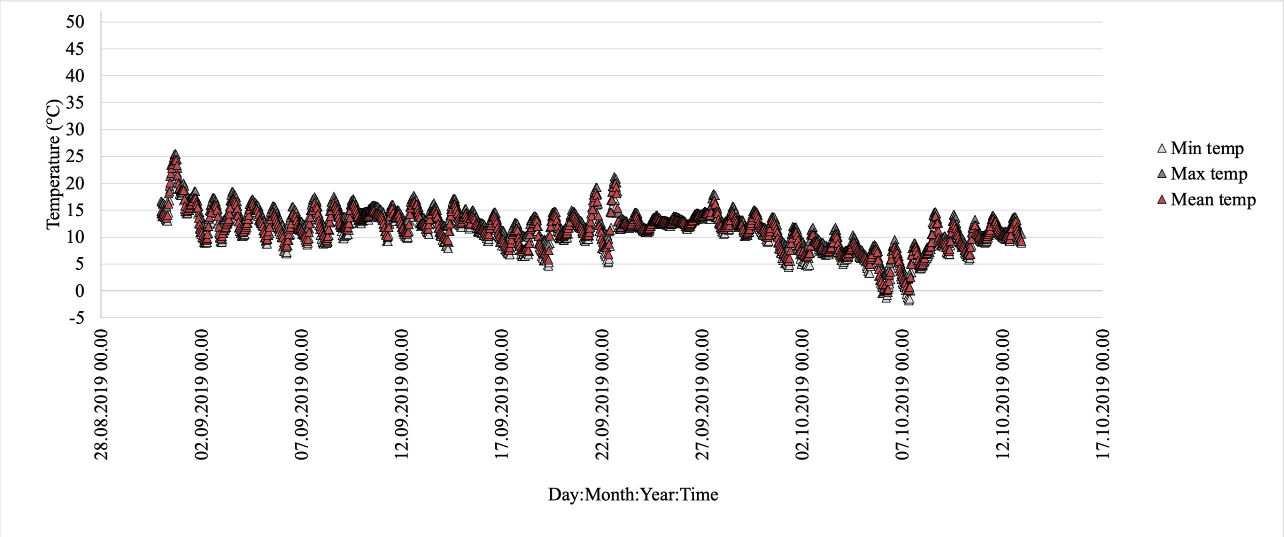

Fig. H. Hourly minimum, mean and maximum temperature (°C) during the time of data collection from the region covering the two study sites in Denmark 31.08.2019-12.10.2019 (Day:Month:Year). The data was attained from the Danish Meteorological Institute (www.DMI.dk) from the region Mariagerfjord municipality.

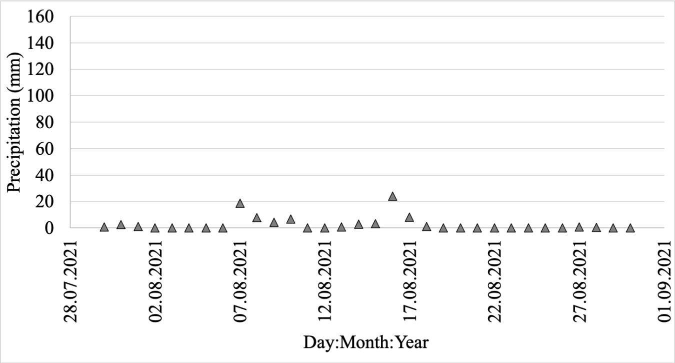

Fig. I. Daily precipidation (mm) during the time of data collection from the region covering the two study sites in Denmark 30.07.2021-30.08.2021 (Day:Month:Year). The data was attained from the Danish Meteorological Institute (www.DMI.dk) from the region Mariagerfjord municipality.

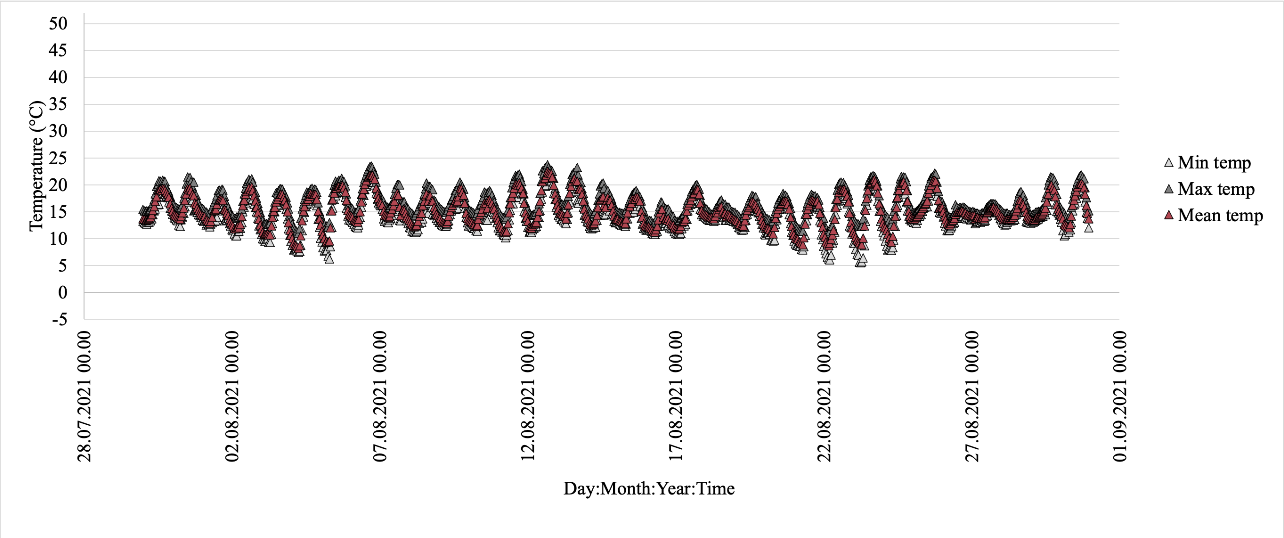

Fig. J. Hourly minimum, mean and maximum temperature (°C) during the time of data collection from the region covering the two study sites in Denmark 30.07.2021-30.08.2021 (Day:Month:Year). The data was attained from the Danish Meteorological Institute (www.DMI.dk) from the region Mariagerfjord municipality.
